# Development of a Novel Engineered Antibody Format for PSMA-Targeted Radionuclide Therapy

**DOI:** 10.1021/acs.molpharmaceut.4c01193

**Published:** 2025-05-22

**Authors:** Nicholas L. Fletcher, Zachary H. Houston, Peter G. Chandler, Eddie Yan, Rob Holgate, Michael Wheatcroft, Kristofer J Thurecht

**Affiliations:** † Centre for Advanced Imaging, Australian Institute for Bioengineering & Nanotechnology, 1974 University of Queensland, St. Lucia, Brisbane, Queensland 4072, Australia; ‡ ARC Research Hub for Advanced Manufacture of Targeted Radiopharmaceuticals, Brisbane, Queensland 4072, Australia; § 664767 Telix Pharmaceuticals Limited, North Melbourne, Victoria 3051, Australia; ∥ Abzena Limited, Cambridge CB22 3AT, United Kingdom

**Keywords:** antibody engineering, pharmacokinetics, targeted therapy, PSMA, prostate cancer, PET imaging

## Abstract

Prostate cancer remains a prevalent and lethal malignancy across the globe. Despite ongoing advances in therapeutic approaches, these remain ineffective, and new treatments are drastically needed. Prostate-specific membrane antigen (PSMA)-targeted radionuclide therapy is a well-developed approach for prostate cancer treatment; however, current small molecule and antibody carriers for molecular radiotherapy each have drawbacks in their biodistribution and consequent side effects as highlighted in current clinical trials. To address this, we developed an approach to bioengineer the well clinically validated antibody carrier HuJ591 to yield an engineered, full-length antibody construct that achieves the beneficial fast pharmacokinetic profile of small molecule carriers alongside the enhanced tumor targeting and reduced renal toxicity of antibody carriers. We report here a rational design process to produce a novel humanized PSMA-targeting antibody designed for the delivery of radiation with abrogated FcRn recycling that aims to reduce blood circulation time and minimize systemic exposure. We demonstrate that these IgG-based constructs retain the favorable properties of HuJ591, such as inherent protein stability, expression in systems compatible with industrial manufacture, and comparable, highly specific PSMA-binding characteristics. We then radiolabeled constructs with the diagnostic radionuclide ^64^Cu as a surrogate for therapeutic radionuclide payloads and undertook a proof-of-concept preclinical imaging study to probe the resulting *in vivo* behaviors. This demonstrated the success of this design strategy to yield the intended *in vivo* and radiopharmaceutical characteristics, with the resulting construct being rapidly cleared from circulation over 3 days. Together, this study demonstrates the rational design of a novel targeting antibody platform for PSMA-expressing tumors with reduced systemic exposure. Such a platform is extremely promising for future radiotherapeutic delivery approaches, whereby effective tumor treatment can be achieved while mitigating potential hematologic toxicity observed with standard antibody delivery approaches.

## Graphical abstract

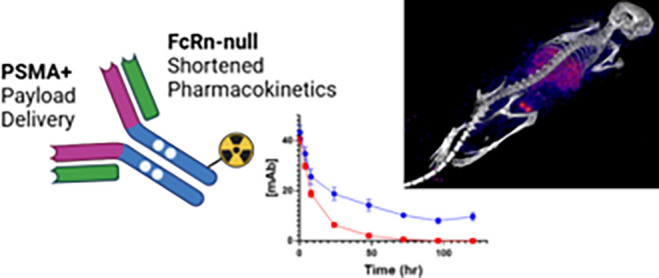

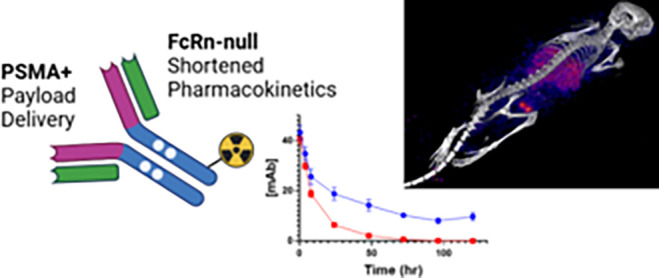

## Introduction

Cancer remains a prevalent disease and ranks as a leading cause of death across the globe, regardless of country’s developmental level.^1^ Prostate cancer in particular is the second most common male cancer, and while there have been advances in both diagnostic and therapeutic approaches, this disease remains responsible for more than 395,000 annual deaths globally.^1^ While often temporarily controlled in the early stage by androgen deprivation therapy or surgical and radiotherapy interventions, the observed high mortality rate demonstrates the limitations of conventional approaches due to resistance or dose-limiting toxicities.^2^ These factors together demonstrate the need for next-generation approaches to treat this deadly and prevalent disease.

Targeted therapies, in particular targeted radionuclide therapy (TRT), offer a unique opportunity to overcome many shortcomings of existing therapeutic approaches.^3^ These approaches utilize antibodies or small molecules, which recognize and bind to cell surface receptors overexpressed on the disease target to selectively deliver therapeutic payloads to the disease site.^4^ One of the key targets with success in this space is prostate-specific membrane antigen (PSMA), which is overexpressed in the majority of prostate cancers, making this an appealing disease target for both imaging and therapy.^5^

There have been multiple notable successes in targeting PSMA for prostate cancer diagnostics and therapeutics. On the diagnostic front, the clinically approved radiolabeled small-molecule peptide ligand [^68^Ga]-PSMA-11 (Gallium Ga-68 gozetotide; Illuccix) is extensively used for positron emission tomography (PET) imaging of PSMA-positive prostate cancer lesions. This enables not just diagnosis but also high-resolution monitoring of the disease to aid clinical decision-making and personalization of appropriate treatment regimens, whereby PSMA-positive patients may be indicated for PSMA-targeted radionuclide therapy.

In terms of PSMA-positive prostate cancer therapeutics, [^177^Lu]-PSMA-617 (lutetium Lu-177 vipivotide tetraxetan; Pluvicto) is approved in a number of countries around the world, and there are many promising TRT approaches in early and late stages of clinical development. [^177^Lu]-PSMA-617 is a radiolabeled small-molecule peptide ligand, which binds to PSMA-positive cells and, as expected of a small molecule, is rapidly cleared from the body by renal elimination – whereby much of the radiation is lost during first-pass clearance, and approximately 50% of the administered dose is eliminated via urine within the first 48 h.^6^ Despite the approval of Pluvicto as a first-generation PSMA therapy, such small molecule-targeting ligands suffer from several drawbacks, including retention in healthy tissues expressing endogenous PSMA, such as lacrimal and salivary glands, neuroendocrine cells in colonic crypts and celiac ganglia, as well as significant potential nephrotoxicity during renal clearance, resulting in undesirable side effects in treated patients.^7,8^

While small molecule ligands offer some favorable characteristics for TRT, the use of antibodies to localize therapeutic payloads to tumor tissues offers greater specificity to tumors vs healthy organs and a highly desirable “platform” for the delivery of targeted radionuclide therapy. PSMA-targeting antibody-delivered radiation has been extensively explored in the clinic for prostate cancer^9^ using the antibody HuJ591. HuJ591 was derived from a murine antibody (J591) using a process of deimmunization to reduce potential immunogenicity. It has been utilized for radioimmunotherapy to deliver radioisotopes such as ^177^Lu and has demonstrated excellent efficacy profiles in multiple clinical trials.^10–12^ HuJ591 is undergoing clinical evaluation in a Phase 3 study as [^177^Lu]-rosopatamab tetraxetan (TLX591) run by Telix Pharmaceuticals (NCT06520345), as well as for the delivery of ^225^Ac in a Phase 2 study as [^225^Ac]-rosopatamab tetraxetan ([^225^Ac]-CONV-01-α) run by Convergent (NCT06549465).

The efficacy of anti-PSMA antibodies as radiotherapeutic carriers is in part boosted by the rapid internalization of the PSMA-bound antibody complex, enhancing payload delivery to achieve high %ID/g and residualizing the radiation within the tumor.^13^ However, at high radiation doses, the long circulation time of antibodies can lead to exposure of the bone marrow that can result in myelosuppression and hematologic toxicity, which represents the primary dose-limiting toxicity for these therapies. The use of dose fractionation, whereby the administered dose is separated into interval doses over time, helps to mitigate the impact and severity of hematologic toxicity while delivering the same amount of radiation to tumor.^14,15^

One appealing approach to generate a next-generation platform for the delivery of radiotherapies is to combine the best attributes of antibodies (cancer selectivity/specificity and residualization of radiation) with those of small molecule carriers (short pharmacokinetic (PK) profile). In this report, we describe just such an approach, achieved through selective engineering to produce a novel, full-length antibody format that specifically targets tumors with radiation while also affecting rapid clearance from the body, with the aim of minimizing dose-related hematologic toxicity. We report the expression and testing of a newly humanized variant of the well-studied and characterized deimmunized HuJ591 antibody, in which the IgG construct incorporates modest, subtle mutations in the Fc domain designed to elicit accelerated clearance from circulation but retain similar PSMA binding and internalization properties of the parent (Figure 1). In this report, preclinical ^64^Cu-PET imaging was used with ^64^Cu as an imaging surrogate for radiotherapeutic payloads to enable sensitive and longitudinal monitoring of construct behaviors. Imaging validated the success of the approach in producing a novel antibody construct, which retained PSMA binding and tumor accumulation with rapid clearance. This engineered PSMA antibody, designated TLX592, is currently undergoing early-stage clinical evaluation for the treatment of prostate cancer.

**Figure 1:**
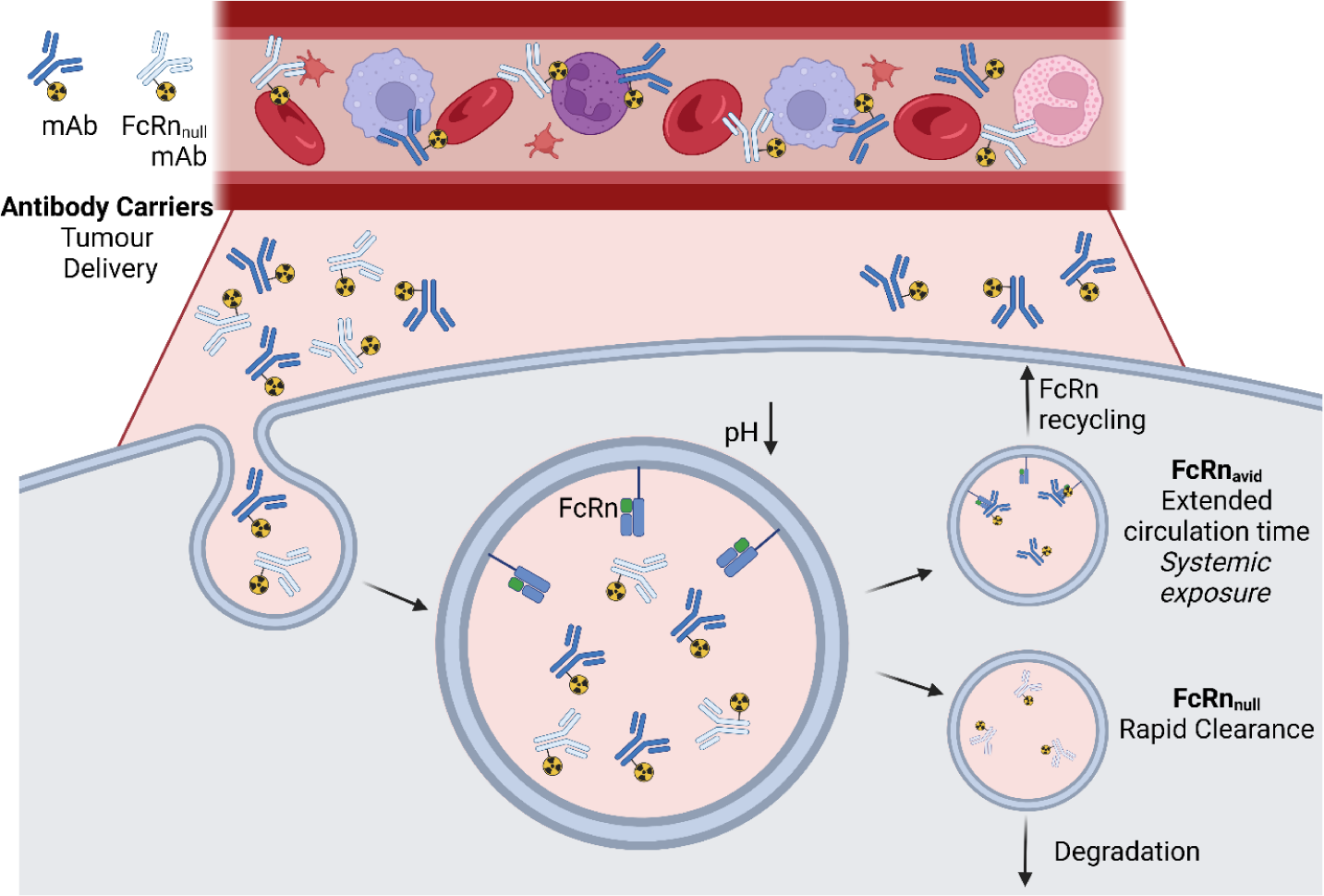
Overarching scheme illustrating FcRn recycling of antibody carriers and the relationship with systemic exposure compared with the design approach taken here. In brief, native IgG antibodies in circulation undergo neonatal Fc receptor (FcRn)-mediated endosomal recycling from catabolic cells, thereby greatly extending their circulation time. When these IgG are then radiolabeled, this results in undesirable systemic exposure. The approach undertaken herein is to use antibody engineering to abrogate IgG FcRn binding and recycling, thereby producing constructs with enhanced clearance rates while maintaining the high specificity and affinity of antibody carriers.

## Materials and Methods

### Antibody Humanization

The murine antibody, mJ591, was humanized using Abzena’s proprietary Composite Human Antibody technology. Composite Human Antibody technology combines structural modeling together with *in silico* technologies to design antibodies with a drastically reduced risk of clinical immunogenicity. Briefly, structural models of the mouse V regions are produced and analyzed in order to identify amino acids that are likely to be important for the PSMA binding properties of the antibody (“constraining residues”). A database of human V region sequences is then analyzed to identify segments of human V region sequences containing each of the constraining residues, which are then combined into a “composite” variable domain sequence. Sequences are then analyzed using *in silico* technologies (e.g., iTope, Abzena’s *in silico* tool for assessing MHC Class II binding), with V region sequences containing predicted nongermline MHC class II binding sites discarded. This process is repeated, culminating in a series of humanized heavy and light chain V region sequences designed from segments of human V region sequences with the objective of avoiding T cell binding epitopes. For the murine J591 antibody, a series of 20 humanized variants were designed and expressed in-house. Variants were characterized in a range of assays, including binding, internalization, and immunogenicity assessment, following which the variant ANT4044 (with CDR sequences complementary to HuJ591, as shown in Figure S1) was designated as the lead candidate to progress for further antibody engineering in this study.

### Modified Antibody Engineering and Production

The VH and VK sequences of the humanized antibody ANT4044 were used to generate a series of dual IgG expression vectors encoding unmodified IgG1, a modified IgG1 (H310A, H435Q), or a modified IgG4 (S228P, L235E, H310A, H435Q) (Figure S2). All constructs were confirmed by DNA sequencing. Constructs are numbered according to the EU numbering scheme.

Endotoxin-free DNA corresponding to the three antibody constructs was prepared and transiently transfected into CHO cells using a MaxCyte STX electroporation system (MaxCyte Inc., Gaithersburg, USA). Following recovery, cells were diluted in CD OptiCHO medium (Thermo Fisher, Loughborough, UK) containing 8 mM l-glutamine (Thermo Fisher, Loughborough, UK) and 1× hypoxanthine-thymidine (Thermo Fisher, Loughborough, UK). 24 h post-transfection, the culture temperature was reduced to 32 °C, and 1 mM sodium butyrate (Sigma, Dorset, UK) was added. Cultures were fed daily by the addition of 3.6% (of the starting volume) feed (2.5% CHO CD Efficient Feed A (Thermo Fisher, Loughborough, UK), 0.5% Yeastolate (BD Biosciences, Oxford, UK), 0.25 mM GlutaMAX (Thermo Fisher, Loughborough, UK), and 2 g/L glucose (Sigma, Dorset, UK)). IgG supernatant titers were monitored by IgG ELISA,and transfected cells were cultured for up to 14 days prior to harvesting supernatants. IgGs were purified from harvested supernatants using either Protein A (IgG1) or Protein G (IgG1 (H310A, H435Q) and IgG4 (S228P, L235E, H310A, H435Q)) Sepharose columns (GE Healthcare, Little Chalfont, UK). All antibodies were buffer-exchanged into 1× PBS at pH 7.2 prior to running on a HiLoad 26/600 Superdex 200 pg preparative SEC column (GE Healthcare, Little Chalfont, UK) using 1× PBS as the mobile phase. Monomeric fractions were collected, pooled, and filter-sterilized, and antibodies were quantified by measuring the OD at 280 nm and using extinction coefficients (Ec(0.1%)) based on their predicted amino acid sequences. Purified antibodies were subsequently analyzed by nonreducing and reducing SDS-PAGE (Figure S3) and by analytical SEC using a Superdex 200 Increase 10/300 GL analytical column (GE Healthcare, Little Chalfont, UK) and 1× PBS as the mobile phase (Figure S4) to confirm purity.

### Antibody Thermostability

To assess the thermostability of the purified antibodies, melting temperatures (the temperature at which 50% of a protein domain is unfolded) were determined by using a fluorescence-based thermal shift assay. All antibodies were diluted to a working concentration of 100 μg/mL in 1× PBS containing SYPRO Orange (Thermo Fisher, Loughborough, UK) and subjected to a temperature gradient from 25 to 99 °C on a StepOnePlus real-time PCR system (Thermo Fisher, Loughborough, UK) over a period of 56 min (Figure S5). The melting curves were analyzed using protein thermostability software (version 1.2), and melting temperatures (*T*_m_s) were calculated based on first derivative data.

### Comparison of Antibody Epitopes on PSMA

HuJ591 and ANT4044 constructs were subjected to Pepscan linear epitope mapping analysis by Pepscan Presto BV (Netherlands).^16,17^ To reconstruct epitopes of the target molecule Human FOLH1 (PSMA; UniProt Q04609), two libraries of peptide-based mimics of 10- and 15-residue lengths were synthesized using Fmoc-based solid-phase peptide synthesis. An amino-functionalized polypropylene support was obtained by grafting with a proprietary hydrophilic polymer formulation, followed by reaction with *t*-butyloxycarbonyl-hexamethylenediamine (BocHMDA) using dicyclohexylcarbodiimide (DCC) with *N*-hydroxybenzotriazole (HOBt) and subsequent cleavage of the Boc groups using trifluoroacetic acid (TFA). Standard Fmoc-peptide synthesis was used to synthesize peptides on the amino-functionalized solid support by custom-modified JANUS liquid handling stations (PerkinElmer, USA).

The binding of the antibody to each of the synthesized peptides was tested in a Pepscan-based ELISA. The peptide arrays were incubated with the primary antibody solution overnight at 4 °C. To prevent nonspecific interactions, SQ blocking buffer was used. SQ consists of 5% ovalbumin from chicken (Sigma, A5253), 5% horse serum (Gibco, Cat. 26-050-088) and 1% Tween 80 in PBS buffer. After washing, the peptide arrays were incubated with a 1/1000 dilution of an appropriate antibody peroxidase conjugate (SBA) for 1 h at 25 °C. After washing, the peroxidase substrate 2,2’-azino-di-3-ethylbenzthiazoline sulfonate (ABTS) and 20 μL/mL of 3% H_2_O_2_ were added. After 1 h, the color development was measured. The color development was quantified with a charge-coupled device (CCD) camera and an image processing system.

The values obtained from the CCD camera range from 0 to 3000 mAU, similar to those of a standard 96-well plate ELISA reader. The results are quantified and stored in the Peplab database. Occasionally, a well contains an airbubble, resulting in a false-positive value, the cards are manually inspected, and any values caused by an airbubble are scored as 0. Epitope candidates are then assessed from peptides showing binding by each antibody to be tested and visualized structurally on a model of the PSMA monomer (AlphaFold: AF-Q04609-F1-v4).

### ANT4044 Antibody PSMA Binding Affinity

Multicycle kinetic analysis was performed on each of the purified antibodies in order to assess binding to PSMA. The analysis was performed using a Biacore T200 instrument running Biacore T200 Evaluation Software V3.0.1 (Uppsala, Sweden). For direct comparison, all antibodies were captured on a Protein G chip (GE Healthcare, Uppsala, Sweden), as the H310A and H435Q mutations abolish Protein A binding. Purified antibodies were diluted to a concentration of 1 μg/mL in HBS-EP+. At the start of each cycle, each antibody was captured on the Protein G surface to give an RL of ∼50 RU. Following capture, the surface was allowed to stabilize. Kinetic data was obtained using a flow rate of 35 μL/min to minimize any potential mass transfer effects. For the kinetic analysis, PSMA (R&D Systems, Minneapolis, USA) was used. Multiple repeats of the blank (PSMA) and a repeat of a single concentration of the analyte were programmed into the kinetic run to check the stability of both the surface and analyte over the kinetic cycles. For kinetic analysis, a 2-fold dilution range was selected from 25 nM to 1.5625 nM PSMA. The association phase of PSMA was monitored for 600 s, and the dissociation phase was monitored for 2400 s. Regeneration of the Protein G surface was conducted using two injections of 10 mM glycine-HCl pH 1.5 containing 0.5% P20 at the end of each cycle.

The signal from the reference channel flow cell Fc1 was subtracted from that of Fc2, Fc3, and Fc4 to correct for differences in nonspecific binding to a reference surface, and a global Rmax parameter was used in the 1-to-1 binding model. The relative *K*_D_ was calculated by dividing the *K*_D_ of each antibody by that of ANT4044 IgG1 on the same chip.

### Antibody FcRn Binding Evaluation

The binding of the purified antibodies to the neonatal receptor; FcRn was assessed by steady-state affinity analysis using a Biacore T200 instrument running Biacore T200 Evaluation Software V3.0.1 (Uppsala, Sweden). Recombinant FcRn protein (Sino Biological, Beijing, China) was coated onto a CM5 chip at 10 μg/mL in sodium acetate at pH 5.5 using standard amine coupling to 300 RU. Purified antibodies were titrated in a five-point dilution from 37 to 3000 nM in PBS containing 0.05% P20 at either pH 6.0 or pH 7.4. Antibodies were passed over the chip in increasing concentrations at a flow rate of 30 μL/min and at 25 °C (Figures S6 and S7). The injection time was 30 s, and the dissociation time was 100 s. Following a single dissociation, the chip was regenerated with 0.1 M Tris at pH 8.0.

### Antibody Fcγ Receptor Binding Evaluation

Binding of purified antibodies to high- and low-affinity Fc gamma receptors was assessed by single-cycle analysis using a Biacore T200 instrument running Biacore T200 Evaluation Software V3.0.1 (Uppsala, Sweden) at a flow rate of 30 μL/min (Table S2 and S3 and Figures S8–14). The human Fc receptors, FcγRI, FcγRIIa (both 167R and 167H polymorphisms), FcγRIIb, FcγRIIIa (both 176F and 176V polymorphisms), and FcγRIIIb, were obtained from Sino Biological (Beijing, China). FcγR was captured on a CM5 sensor chip precoupled using a His capture kit (GE Healthcare, Little Chalfont, UK) using standard amine chemistry. At the start of each cycle, His-tagged Fc gamma receptors diluted in HBS-P+ were loaded to a specific RU level. A five-point, 3-fold dilution range of antibody without regeneration between each concentration was used for each receptor. The RU-loaded, association and dissociation times together with the concentration range used for each receptor are shown in Table S2. In all cases, following dissociation, the chip was regenerated with two injections of glycine pH 1.5. The signal from the reference channel Fc1 (blank) was subtracted from that of the Fc loaded with receptor to correct for differences in nonspecific binding to the reference surface. Sensorgrams were analyzed for 1:1 kinetics for the high-affinity Fc gamma receptor FcγRI and by steady-state binding for the low-affinity Fc gamma receptors.

### Antibody Bioconjugation with DOTA

Each antibody was prepared as a 6 mg/mL solution in 0.1 M NaHCO_3_ and 20 mM ethylenediaminetetraacetic acid (EDTA), pH 8–9 (reaction buffer). Next, 1,4,7,10-tetraazacyclododecane-1,4,7,10-tetraacetic acid mono-*N*-hydroxysuccinimide ester hexafluorophosphate trifluoroacetate salt (NHS-DOTA (Macrocyclics, TX, USA) reagent) was prepared in Dulbecco’s PBS, pH 7.2–7.5, at 5.0 mg/mL. 10–25 equiv of NHS-DOTA reagent was added to the solution, and the antibody concentration was adjusted to 4.0 mg/mL by the addition of reaction buffer. Reactions were incubated at 22 °C for 2–3 h. Next, the reaction was quenched by the addition of 0.2 M sodium acetate, pH 5.5 (10:1 v/v), and ultracentrifuged at 12,000 rcf with Vivaspin filters (30 kDa MWCO, PES membrane, Vivaproducts, MA, USA). The dilution and ultracentrifugation were repeated 3× more. Finally, products were buffer-exchanged 6× into Dulbecco’s PBS, pH 7.2–7.5, with Vivaspin filters (30 kDa MWCO, PES membrane). The antibody-DOTA conjugate concentration was measured by a UV–vis spectrophotometer, corrected to 4.0 mg/mL with Dulbecco’s PBS pH 7.2–7.5, sterile-filtered (0.22 μm cellulose acetate filters), and stored at −80 °C.

### IgG-DOTA Bioconjugate LC–MS Analysis

LC–MS analysis was carried out using a Waters XEVO G2S TOF mass spectrometer connected to an Acquity H-Class UPLC system (Waters, USA) with a Poroshell 300SB-C3 guard, 5 μm RP column (Agilent, USA). The mobile phase was Buffer A (0.1% formic acid in water). A gradient (2.5 min 10% B, 10–80% B gradient in 3.5 min, re-equilibration 10% B 3.5 min) was applied using Buffer B (MeCN–0.1% formic acid) at a flow rate of 0.6 mL/min. Samples were deglycosylated with GlycINATOR (from Genovis) prior to analysis. GlycINATOR 2000 units (Genovis, Sweden) were reconstituted with 50 μL of double-distilled endotoxin-free water. The IgG-DOTA conjugates were diluted to 0.4 mg/mL with Dulbecco’s PBS, and a sample of 50 μL (20 μg) of each conjugate was incubated with 1 μL (40 units) of GlycINATOR solution at 37 °C for 1 h. A 10 μL injection of a 0.4 mg/mL sample of each deglycosylated conjugate was then analyzed (Figures S15–17 panels A).

### IgG-DOTA Bioconjugate SEC Analysis

Analytical SEC was carried out using a TOSOH Bioscience TSKgel Super SW 3000 column (4.6 mm × 30 cm, 4 μm) and a guard column (4.6 mm × 4 cm; Tosoh Bioscience, Japan), connected to a Dionex Ultimate 3000 UPLC system (Dionex, CA, USA). The mobile phase was 0.2 M potassium phosphate buffer, pH 6.8 (0.2 M potassium chloride, 15% isopropanol). The flow rate was kept constant at 0.35 mL/min. The column was maintained at 25 °C throughout the analysis. The analysis was carried out using a 20 min isocratic elution, with UV detection at 280 nm for aggregate analysis and 248 nm for residual reagent analysis (Figures S15–17 panels B). 40 μg of each IgG-DOTA conjugate was injected for aggregate and residual reagent analysis. The percentage purity and percentage aggregation were calculated by comparing the peak areas of the main peaks and early eluting peaks, respectively, with the total peak area.

### DOTA Bioconjugate PSMA Binding

SPR analysis was conducted to compare the binding of HuJ591-DOTA and ANT4044-DOTA to cynomolgus, human, and mouse PSMA (Figure S18 and Table S4). Single-cycle kinetic analysis was performed using purified proteins at 25 °C on a Biacore T200 instrument (Cytiva, Marlborough, USA). HBS-EP+ (Cytiva, Marlborough, USA) was used as the running buffer, as well as for ligand and analyte dilutions.

At the start of each cycle, antibodies diluted in running buffer to 1 μg/mL were loaded onto Fc2, Fc3, and Fc4 of a Protein G capture sensor chip (Cytiva, Marlborough, USA) at a flow rate of 10 μL/min to give an immobilization level (RL) of ∼50 RU. The surface was then allowed to stabilize. Single-cycle kinetic data was obtained using PSMA as the analyte, injected at a flow rate of 40 μL/min to minimize any potential mass transfer effects.

A three-point, 2-fold dilution range from 12.5 to 50 nM of antigen in running buffer was used without regeneration between each concentration. The association phases were monitored for 200 s for each of the three injections of increasing concentrations of antigen, and a single dissociation phase was measured for 1500 s following the last injection of antigen. 10 mM glycine-HCl, pH 1.5 containing 0.5% P20 (Tween 20) was used to regenerate the chip surface. The signal from the reference channel Fc1 (no IgG captured) was subtracted from that of Fc2, Fc3, and Fc4 to correct for bulk effects and differences in nonspecific binding to a reference surface. The signal from each IgG blank run (IgG captured but no antigen) was subtracted to correct for differences in surface stability.

The double-referenced sensorgrams were fitted with the Langmuir (1:1) binding model, where the closeness of fit of the data to the model is evaluated using the Chi-square value, which describes the deviation between the experimental and fitted (observed and expected) curves. The fitting algorithm seeks to minimize the Chi-square value.

### Radiolabeling IgG-DOTA Bioconjugates with ^64^Cu

All antibodies were incubated with ^64^Cu at a 200-fold excess of biomolecule in 0.1 M ammonium acetate buffer, pH 5.5, for 45 min at room temperature. Samples of each solution were taken and mixed 1:1 with 50 mM EDTA. 5 μL of each solution was spotted on thin layer chromatography (TLC) paper (iTLC-SG glass microfiber chromatography paper impregnated with silica gel (Agilent, USA)) and run with 50:50 H_2_O:ethanol. Plates were then imaged on a Carestream MSFX imaging system (Bruker, Australia) using a radioisotopic phosphor screen. Radiolabeled constructs were buffer exchanged into PBS using 7K MWCO Zeba Spin Columns (Thermo Scientific, USA) as per manufacturer’s protocols. All samples showed ≥95% radiopurity prior to injection. Control experiments were conducted to monitor the elution behavior of free [^64^Cu] and [^64^Cu]­EDTA for quality control. A representative radioTLC image is shown in Figure S18, showing that ^64^Cu was bound to the antibodies. All ^64^Cu-labeled constructs used in this study were prepared at 3.04 × 10^11^ Bq/g (4.6 × 10^16^ Bq/mol).

### Radio SEC Analysis of [^64^Cu]­DOTA-Antibody

[^64^Cu]-labeled conjugates were analyzed using Radio-SEC. 20 μL of the reaction mixture was injected into an SEC column (TSK gel G3000SWXL 5 μm, 300 × 7.8 mm, TOSOH, Japan). Analysis was performed in isocratic mode at a fixed flow rate of 0.75 mL/min using PBS, pH 7.4, as the mobile phase. Chromatograms were recorded at 280 nm to detect protein, and radioactivity was detected using a Flow-Count radioisotope HPLC detector (B-FC-3300, Eckert & Ziegler, Germany) attached to a Shimadzu modular system comprising a DGU-405 degasser, an SPD-M40 photodiode array, and an LC-40D solvent delivery module (Shimadzu, Australia) at a signal sensitivity of 200 K. The system was calibrated with appropriate size standards (Gel Filtration Standard Cat# 1511901, Bio-Rad, USA) to confirm the observed monomeric IgG elution profile.

### Tumor Xenograft Model

Healthy male Balb/c nude mice (∼20 g) from 8 weeks old were obtained from the Animal Resource Centre (ARC, Western Australia) and used for this study. Mice were imported into the Centre for Advanced Imaging (CAI) animal holding facility and monitored for 1 week prior to the study in order to acclimate to the environment prior to the injection of cells. All animals were provided with free access to food and water before and during the imaging experiments, which were approved by the University of Queensland Animal Ethics Committee (Approval # AIBN/CAI/530/15/ARC/NHMRC).

Mice were injected (27G) subcutaneously with 5 × 10^6^ LNCaP cells in 100 μL of phosphate-buffered saline into the right flank of each mouse. Tumors were allowed to develop over 1–2 months and enrolled in imaging studies as tumors reached appropriate sizes for imaging (∼100 mm^3^).

### [^64^Cu]­PET Imaging and Biodistribution of Constructs

LNCaP tumor-bearing mice were anesthetized with isoflurane (IsoFlo, Abbott Laboratories, USA) at a dose of 2% in medical O_2_ in a closed anesthetic induction chamber. Mice were monitored by using ocular and pedal reflexes to ensure deep anesthesia. Once the mouse was deeply anesthetized, it was placed on an appropriate animal bed, where the anesthetic air mixture (1%) was delivered to its nose and mouth through a nose cone. The injection syringe was filled with the [^64^Cu]-labeled IgG construct solution (∼150 μL PBS), and the activity in the syringe was measured using a dose calibrator (Capintec CRC-25) with a calibration factor of 35. Anesthetized mice were then administered imaging doses via injection into the lateral tail vein (29G needle). The activity left in the syringe after the tail vein injection was measured using the same dose calibrator, and the total activity injected into each mouse was calculated, with all animals receiving between 2.6 and 3.4 MBq [^64^Cu]-labeled construct.

Two cohorts of mice were administered each of the three engineered [^64^Cu]­DOTA-PSMA antibody variants and the comparator molecule: [^64^Cu]­DOTA-HuJ591. Blood samples were collected from the first cohort (*n* = 4) at 0.5, 8, 24, and 48 h post-administration for *ex vivo* determination of circulating levels in the blood. This cohort was additionally utilized for the PET imaging study below up to 48 h post-administration at which time mice were sacrificed and organs were collected for *ex vivo* gamma counting and determination of biodistribution. Blood samples were collected from the second cohort of mice (*n* = 4 (except HuJ591 where *n* = 3)), for each variant at 0.5, 4, 72, 96, and 120 h post-administration for *ex vivo* determination of circulating levels in the blood. This cohort was then utilized to determine biodistribution profiles of the variants at 120 h post-administration using *ex vivo* gamma counter analysis.

All blood samples were collected by tail snip (∼20–50 μL) directly into gamma counter tubes. The blood samples were then weighed and analyzed using a PerkinElmer 2480 Automatic Gamma Counter, with calibration and calculation of %ID/g as described below.

At prescribed times following [^64^Cu]­DOTA-IgG injection, mice were again anesthetized and placed on the scanner bed (*n* = 4 per scan using a bed developed in-house). Physiological monitoring (respiratory using a sensor probe) was achieved throughout all experiments using an animal monitoring system (the BioVet system, m2m Imaging, Australia). Images were acquired by using a Siemens Inveon PET-CT scanner. Micro-CT scans were first acquired for anatomical coregistration. The CT images of the mice were acquired through an X-ray source with the voltage set to 80 kV and the current set to 500 μA. The scans were performed by using 360° rotation with 120 rotation steps, with a low magnification and a binning factor of 4. The exposure time was 230 ms, with an effective pixel size of 106 μm. The total CT scanning process took approximately 15 min. The CT images were reconstructed by using Feldkamp reconstruction software (Siemens). Following CT imaging, PET scans were acquired at 8, 24, and 48 h after injection of the radiotracer, using 30–60 min static acquisitions. The PET images were reconstructed using an ordered-subset expectation maximization (OSEM2D) algorithm and analyzed using the Inveon Research Workplace software (IRW 4.1) (Siemens), which allows fusion of CT and PET images and definition of regions of interest (ROIs). CT and PET data sets of each individual animal were aligned using IRW software (Siemens) to ensure good overlap of the organs of interest. Three-dimensional ROIs were placed within the whole body, as well as all the organs of interest, such as the heart, kidney, lungs, bladder, liver, spleen, intestines, and tumor, using morphologic CT information to delineate organs. Activity per voxel was converted to nCi/cc using a conversion factor obtained by scanning a cylindrical phantom filled with a known activity of ^64^Cu to account for PET scanner efficiency. Activity concentrations were then expressed as percentage of the decay-corrected injected activity per cm^3^ of tissue that can be approximated as percentage injected dose/gram (%ID/g).

For all *ex vivo* gamma counter biodistribution studies, blood was sampled, and tissues were collected and cleaned of excess blood and weighed in appropriate gamma counter tubes. A PerkinElmer 2480 Automatic Gamma Counter (PerkinElmer, USA) was used to measure radioactivity in all tissue samples. The gamma counter was calibrated using known samples of ^64^Cu, and measured activity was presented as %ID/g based on injected activities.

### Statistical Analysis

Statistical analysis was performed using GraphPad Prism 10. Unpaired, parametric, two-tailed *t*-tests were used to compare the distribution of each construct within each tissue measured. Levels of significance are reported in the figure captions.

Measured circulating levels of [^64^Cu]-labeled constructs over 120 h were then fitted to a single-phase exponential decay model (GraphPad Prism 10) to determine the biological half-lives of each variant.

### DOTA-Conjugate Stability

Lead compound DOTA-functionalized IgG1 (H310A, H435Q) (PK-modified anti-PSMA IgG) was assessed for stability in a range of formulations. The conjugate at 10 mg/mL was prepared in 20 mM Na acetate buffer with the addition of 150 mM NaCl or 250 mM Na acetate buffer and compared to PBS (pH 7.1–7.5) alone. Samples were prepared and then stored at −20 °C, 25 °C, or 40 °C. Sample concentration was assessed by absorbance at 280 nm (OD280) and analyzed by analytical SEC after 2 or 4 weeks of storage or after 3 cycles of freeze–thaw. No change in sample concentration determined by absorbance readings was observed under any conditions. Analytical SEC was undertaken using Acquity UPLC Protein BEH 200 (Waters, USA), 4.6 mm i.d. × 15 cm, 1.7 μm; 0.2 M KCl, 0.2 M K_2_HPO_4_, pH 6.8, 15% isopropanol (v/v); elution: isocratic, 10 min; 0.35 mL/min; 30 °C; 10 μg injection. Results are presented as % High Molecular Weight (HMW), % monomer, and % Low Molecular Weight (LMW) fragments, shown in Table S4.

## Results and Discussion

### Humanized Anti-PSMA Antibodies

The basis of the antibody engineering described in this work was the generation of the novel humanized PSMA-targeting antibody, ANT4044. The mJ591 antibody was used as the starting point for this work, as this parent antibody, along with the historically deimmunized variant Hu591, is a well-established targeting ligand for PSMA that has demonstrated efficacy in numerous clinical studies.

The PSMA-targeting ANT4044 construct was derived from mJ591 sequences by Abzena using their proprietary composite human antibody humanization processes (as described in Materials and Methods). Significantly, ANT4044 maintained sequences in the complementarity-determining regions (CDRs), as well as framework residues (Figure S1) compared to mJ591 or HuJ591. The resulting humanized clone was evaluated using iTope, a proprietary (Abzena) technology for *in silico* analysis of peptide binding to human MHC class II alleles (SI section 1.2).

The results showed that VH and Vκ domains for ANT4044 had better iTope scores than the existing Hu591 sequences (Table 1), suggesting reduced immunogenicity following the design process, which is desirable for antibody-targeting platforms.

**Table 1:** A Summary of iTope Scores for Humanized Heavy-Chain and Light-Chain Variable Region Sequences for ANT4044 in Comparison to the Equivalent HuJ591 Sequence^a^

Predicted affinity MHC class II	HuJ591 VH	ANT4044 VH
Heavy Chain		
High	3	1
Low	4	1

Predicted affinity MHC class II	HuJ591 Vκ	ANT4044 Vκ
Light Chain		
High	1	1
Low	4	5

a Numbers represent the number of T-cell epitopes in the sequence based on either High or Low stringency.

### Antibody Engineering for PK Modulation

The humanized construct ANT4044 was subjected to a series of engineering steps with the aim of incorporating these novel PSMA-targeting domains into a platform with a reduced PK profile to achieve the desired reduction in systemic exposure. IgG binding of the neonatal Fc receptor (FcRn) is associated with greatly extended circulation times due to endosomal FcRn recycling in catabolic cells (Figure 1).^18^ Inhibition of this interaction is therefore an attractive approach to reducing the PK profile of such constructs. Three variants were generated (Figure 2), dual IgG expression vectors encoding unmodified IgG1, IgG1 harboring the mutations H310A and H435Q (that abolish FcRn binding,^19^ referred to as IgG1 (H310A, H435Q)), and a modified IgG4 with the same FcRn-abolishing mutations described above, together with the hinge-stabilizing S228P mutation^20^ and the Fc-silencing L235E mutation^21^ (referred to as IgG4 (S228P, L235E, H310A, H435Q)).

**Figure 2:**
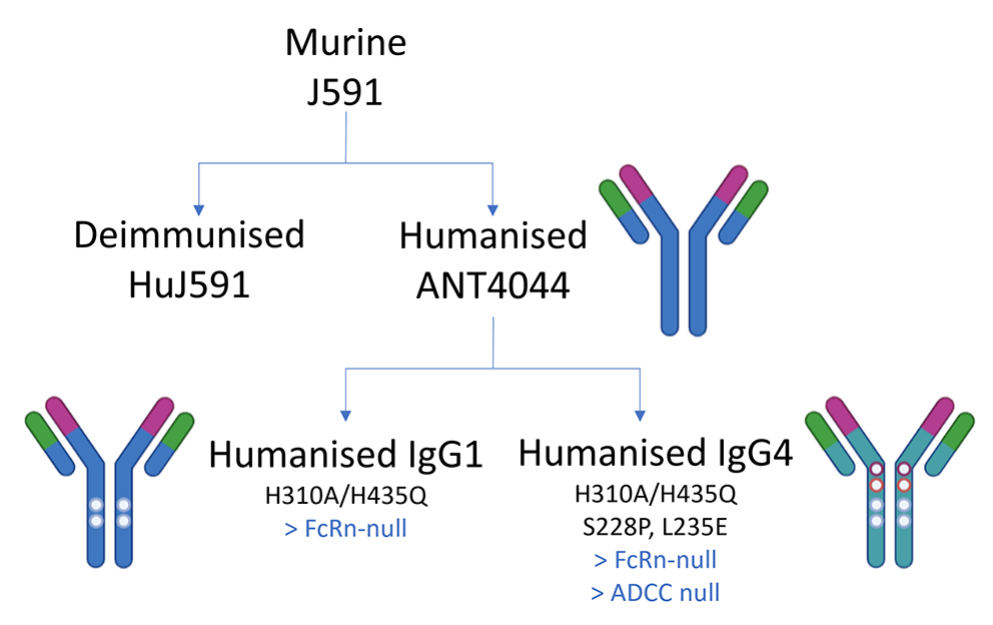
Diagram showing the relationship of PSMA-binding antibodies described in this work including targeted engineering of ANT4044 variants to abolish FcRn and Fc interactions to abrogate FcRn-mediated recycling and antibody-dependent cellular-cytotoxicity (ADCC) mechanisms, respectively.

As anticipated, the affinity of Protein A to bind antibodies was impaired in the antibodies harboring FcR mutations; however, Protein G was employed effectively for affinity purification. Constructs were expressed in CHO cells and purified using either Protein A (IgG1) or Protein G (IgG1 (H310A, H435Q) and IgG4 (S228P, L235E, H310A, H435Q)) chromatography, followed by preparative size-exclusion chromatography. The integrity of the purified antibody constructs was characterized by both nonreducing and reducing SDS-PAGE that showed bands corresponding to the predicted sizes of intact IgG and heavy and light chains, respectively (Figure S3) and by analytical SEC that showed elution times consistent with correctly folded monomeric IgG species (Figure S4), in both cases congruent with the production and purification of designed and folded constructs with the correct size.

### Antibody Thermostability

The thermostability of all three purified constructs was assessed using a fluorescence-based thermal shift assay to determine the *T*_m_ (“melting temperature” at which the protein is 50% unfolded) of each construct (Figure S5 and Table S1). The analysis was able to fit 2–3 discrete *T*_m_ transitions for each construct, congruent with the multiple and discrete domains of an IgG,^22^ as the unfolding of the Fab and the Fc are often considered to be independent events. The *T*_m2_ and *T*_m3_ profiles are broadly comparable between IgG1 and IgG1 (H310A, H435Q) (consistent with the Fab portion being identical), while *T*_m1_ is slightly lower for IgG1 (H310A, H435Q) (*T*_m1_ values of 69 and 65 °C, respectively). However, both IgG1 variants are more stable than the IgG4 variant (*T*_m1_ values of 59 °C and *T*_m2–3_ lower than IgG1), as has previously been reported for IgG1/IgG4 with the same variable regions.^23^

### PSMA Binding

The goal of this work was to produce PK-modified humanized antibody constructs for targeting PSMA-expressing lesions; therefore, retention of PSMA binding in the generated constructs is of key importance. To first probe this, Pepscan analysis was used to compare the putative binding epitopes of the newly generated ANT4044 construct in comparison to HuJ591 (Figure 3). Three discontinuous epitopes were identified within the library of PSMA peptides, covering the sequences 155-SDIVPPFSAFSP, 244-DGWNLPGGGVQRGNILN, 311-GSAPPDSSWRGSLKVPY, and 327-YNVGPGFTGNFSTQK, with a high degree of congruence between antibodies (>89%). While discontinuous within the linear antibody sequence, when visualized on the folded protein conformation (Figure 3), these peptides form a continuous epitope surface shared by both antibodies. This similarity in predicted binding sites is consistent with the design approach utilized here to produce PSMA-binding constructs based on the binding domains of mJ591.

**Figure 3:**
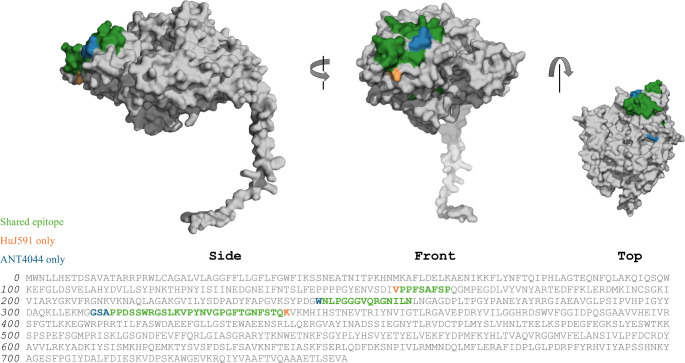
Visualization of Pepscan analysis to identify putative epitopes of PSMA bound to HuJ591 and ANT4044. For visual comparison of the epitopes bound by each antibody, identified epitopes are identified on a model of the PSMA monomer (AlphaFold: AF-Q04609-F1-v4).

To further confirm that the resulting constructs retained their binding efficacy, ANT4044 and all derived constructs were assessed for affinity to PSMA by surface plasmon resonance (SPR) through multicycle kinetic analysis on a Biacore system (Figure 4A). All three ANT4044 variants showed high-affinity binding to PSMA, and calculated parameters from the sensorgram data (Table 2) show negligible variation in measured *K*_D_ (∼700 pM) for all constructs, comparable to the parent antibody, which is consistent with, or slightly improved over, previously values reported for mJ591.^24,25^ This suggests that reformatting ANT4044 variable domains into different constant domains has minimal impact on target antigen binding, as is desirable for their intended application.

**Figure 4:**
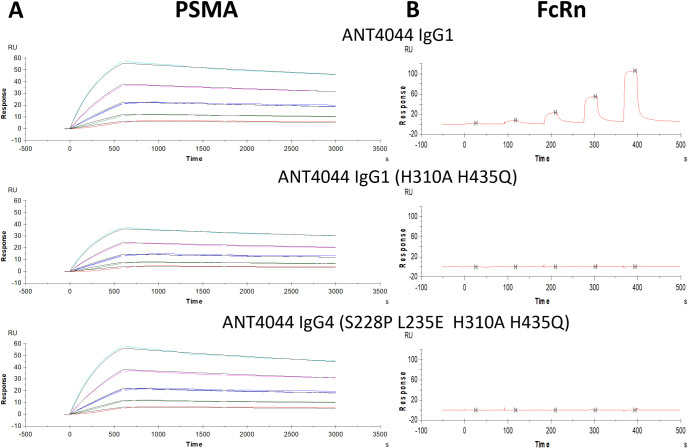
ANT4044 IgG variants binding profiles. (A) Representative multicycle sensorgram data and fitted curves demonstrating PSMA affinity of all constructs (colored lines; raw data for PSMA applied over a 2-fold dilution range from 25 nM (teal) to 1.5625 nM (red), black lines; fitted data). (B) Steady state affinity sensorgrams of the binding of constructs to FcRn at pH 6.0.

**Table 2:** Multicycle Kinetic Data for the Binding of ANT4044 IgG Variants to PSMA, as Determined Using Surface Plasmon Resonance

IgG Construct	*K*_a_ (1/Ms)	*K*_d_ (1/s)	*K*_D_ (M)	Chi^2^ (RU^2^)	*K*_D_ Relative to ANT4044
ANT4044 IgG1	1.07 × 10^5^	7.88 × 10^–5^	7.34 × 10^–10^	0.507	1.00
IgG1 (H310A, H435Q)	1.01 × 10^5^	7.48 × 10^–5^	7.37 × 10^–10^	0.421	1.00
IgG4 (S228P, L235E, H310A, H435Q)	1.39 × 10^5^	1.04 × 10^–4^	7.43 × 10^–10^	0.548	1.01

### FcRn Binding

The novel aim of this antibody engineering approach was to abrogate the FcRn binding of the antibody constructs to produce shortened PK profiles via faster clearance from the blood. The binding of purified ANT4044 IgG constructs to FcRn was assessed by steady-state affinity analysis on a Biacore system (Figure 4B). Analysis of the binding data showed that IgG1 affinity for FcRn was measured to be 1.3 μM at pH 6.0, while increasing pH to 7.4 inhibited this interaction, as expected for a native IgG-FcRn interaction. As anticipated from the targeted Fc mutations, neither IgG1 (H310A, H435Q) nor IgG4 (S228P, L235E, H310A, H435Q) displayed measurable binding to FcRn at either pH, congruent with the successful inhibition of the FcRn interaction. This supports the successful design of PSMA-binding constructs, which will not display prolonged circulation due to FcRn recycling.

### Fcγ Receptor Binding

Similarly to the FcRn analysis, the affinity of the designed constructs for Fcγ receptors was assessed by utilizing SPR on a Biacore system. Representative sensorgrams are shown in Figures S8–14 with analysis summarizing these findings in Table S3. In short, the ANT4044 unmodified IgG1 construct bound to all high- and low-affinity human activating Fcγ receptors as expected, and the introduction of H310A and H435Q mutations did not inhibit these interactions. As expected, given the design process, the IgG4 (S228P, L235E, H310A, and H435Q) construct showed markedly reduced binding to all activating Fcγ receptors. These data are congruent with the intended point mutations, whereby IgG1 and IgG1 (H310A, H435Q) retain wild-type effector function, while IgG4 (S228P, L235E, H310A, H435Q), which shows reduced affinity for the involved receptors, will have impaired effector function.

These results are consistent with the successful design and production of PSMA-binding constructs with modulated PK profiles. Of the novel constructs produced, IgG1 (H310A, H435Q) is of great potential interest for targeted therapeutic delivery while retaining effector function and is the key focus of the remainder of these studies. However, the further-engineered IgG4 (S228P, L235E, H310A, and H435Q) may have potential applications where this effector functionality is undesirable. We next examined the behavior of these constructs in more complex biological environments to assess the success of engineering tumor targeting and PK profiles.

### Chelator Functionalization and [^64^Cu] Labeling

Preclinical positron emission tomography (PET) imaging was employed to study the construct behaviors *in vivo,* as this modality allows quantitative imaging of labeled material behaviors isotropically throughout the body and is readily clinically translatable.^26^ ^64^Cu was chosen for this study as the radioactive half-life of 12.7 h enables preclinical PET imaging and *ex vivo* biodistribution analysis for several days, which was anticipated to be sufficient for the fast-clearing antibodies engineered herein. This requires the incorporation of a radiometal-binding ligand in the targeted construct. In this case, the macrocycle 1,4,7,10-tetraazacyclododecane-1,4,7,10-tetraacetic acid (DOTA) was chosen as a chelator, as this ligand allows binding of the PET radioisotope ^64^Cu while also allowing potential future translation to radiotherapeutic isotopes such as ^177^Lu.^27^ While DOTA is not the optimal chelator for ^64^Cu compared with some other macrocyclic chelators available,^28^ it was chosen to enable the same chelator-antibody construct to be utilized for future radiotherapy studies without introducing potential variability by altering the chelator used.

The amine-reactive species DOTA-NHS was utilized to functionalize the IgG constructs (Figure 5A). Following purification to remove unbound chelator, we used analytical LC–MS (Figures 5B and S15–17) to determine the average drug to antibody ratio (DAR) for each conjugate and showed all to achieve 4.0–4.9 DOTA per IgG (Table 3). Subsequent analytical SEC (Figures S15–17) confirmed that all species retained their monomeric structure (Table 3). To confirm that DOTA functionalization did not impact target antigen binding, ANT4044-DOTA and control HuJ591-DOTA were assessed for PSMA binding by SPR analysis (Figure S18 and Table S4). This showed both constructs to possess equivalent affinity for cynomolgus and human PSMA in the low nanomolar range, with no measurable binding to murine PSMA. This is congruent with the design of ANT4044 to retain PSMA binding properties similar to HuJ591 and confirms that DOTA bioconjugation does not significantly impact this binding affinity.

**Figure 5:**
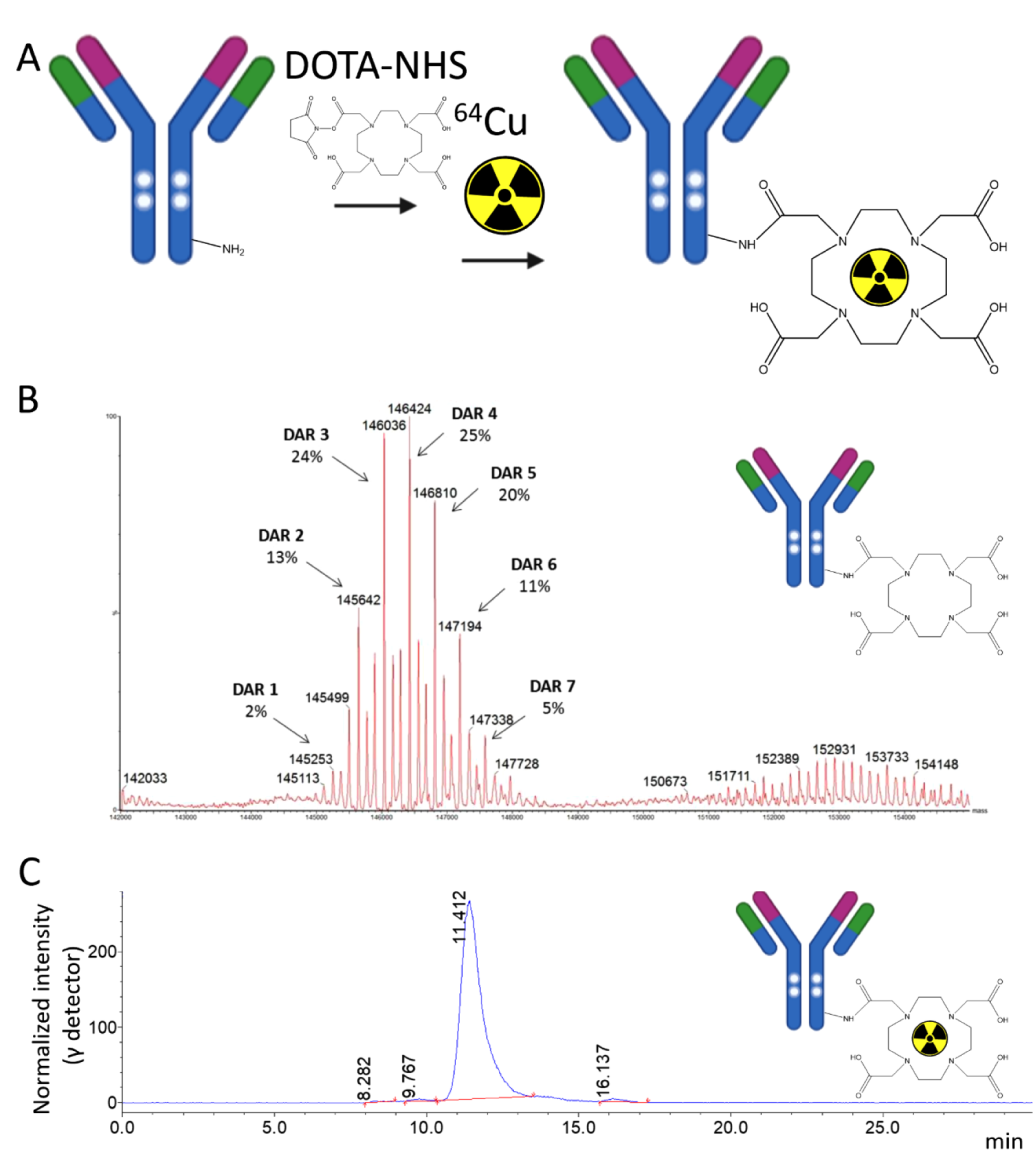
ANT4044 IgG1 (H310A, H435Q) functionalization for preclinical ^64^Cu PET imaging. (A) Schematic outline of IgG DOTA and ^64^Cu radiolabeling for preclinical PET imaging. (B) LC–MS spectrum for ANT4044 IgG1-DOTA (H310A, H435Q) to determine DOTA DAR. (C) Radio-SEC chromatogram of [^64^Cu]­ANT4044 IgG1-DOTA (H310A, H435Q), demonstrating the effective radiolabeling of monomeric IgG species.

**Table 3:** Determined DOTA Degree of Functionalization on ANT4044 Species and Monomer Purity

IgG Construct	DAR (LC–MS)	% monomer (SEC)
ANT4044 IgG1	4.4	98.9
IgG1 (H310A, H435Q)	4.0	99.0
IgG4 (S228P, L235E, H310A, H435Q)	4.9	98.7

DOTA constructs were then [^64^Cu]-labeled, achieving >95% radiopurity as assessed by radio-TLC. Analytical radio-SEC was then used to confirm effective [^64^Cu]-labeling of monomeric IgG species (Figure 5C) prior to preclinical imaging.

### Antibody *In Vivo* Behavior

The effect of targeted antibody engineering approaches on the pharmacokinetic profile and biodistribution of ANT4044 IgG variants was then assessed in a preclinical *in vivo* study. [^64^Cu]-labeled constructs were administered to LNCaP (PSMA+) xenograft tumor-bearing male Balb/c nude mice, and preclinical [^64^Cu]­PET in combination with *ex vivo* gamma counting of tissues was used to monitor the construct PK and biodistribution in comparison to [^64^Cu]-labeled HuJ591 as a previously well-studied reference antibody. At 48 h post-administration, PET imaging showed the lead construct ANT4044 IgG1 (H310A, H435Q) to be well distributed throughout the body, with significant accumulation in the tumor and clear hepatic clearance (Figure 6A). *Ex vivo* quantitation of construct biodistribution at the same time point was undertaken and showed marked differences in biodistribution between constructs (Figure 6B). Unmodified constructs ANT4044 IgG1 and HuJ591 follow a distribution profile expected for a tumor-specific IgG, with enhanced accumulation in the tumor and significant levels remaining circulating in the blood. In comparison, both PK-engineered antibody constructs display significantly reduced levels in the blood (<30% of the level of unmodified constructs observed at the 24 h time point) yet display comparable levels in the tumor. Unsurprisingly, the reduced levels of PK-engineered variants in the blood are associated with elevated levels in radio-tolerant clearance organs (liver and spleen), in congruence with the rapid clearance from circulation and expected clearance pathways of non-FcRn recycling antibodies.

**Figure 6:**
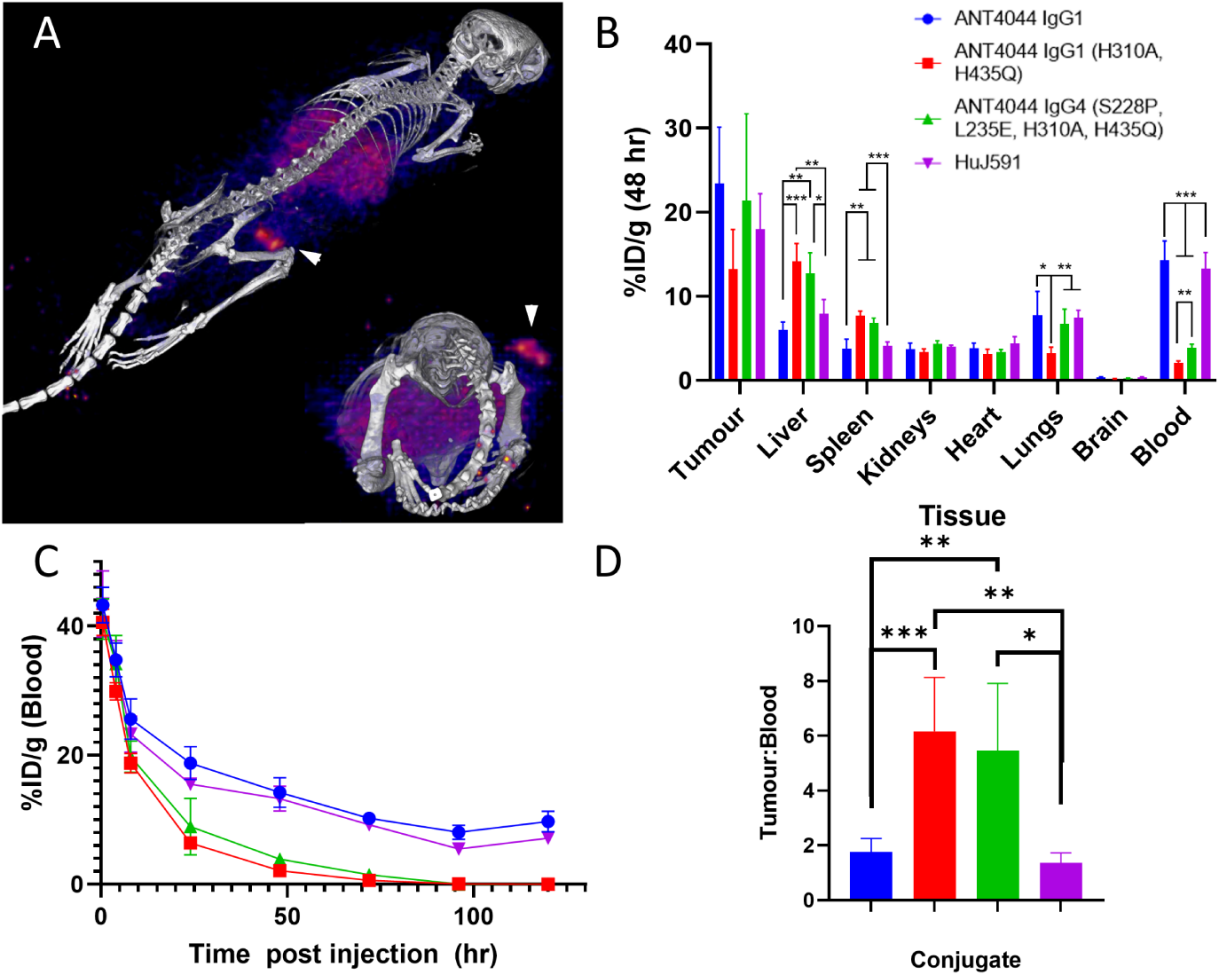
Preclinical biodistribution and PET imaging of [^64^Cu]-labeled constructs in an LNCaP xenograft model. Color profiles of all plots were as per legend in (B). (A) Exemplar PET-CT image of ANT4044 IgG1 (H310A, H435Q) biodistribution at 48 h post-administration (arrow highlights tumor). (B) *Ex vivo* biodistribution of all ANT4044 constructs in comparison to HuJ591 at 48 h post-administration. (C) Blood profile of ANT4044 constructs in comparison to HuJ591 over 120 h post-administration. (D) Tumor:Blood ratio of ANT4044 constructs in comparison to HuJ591 at 48 h post-administration. * Denotes *p* < 0.05, ** Denotes *p* < 0.01 and *** Denotes *p* < 0.001 via unpaired *t* test. Where no significance level is indicated, differences between groups were not statistically significant.

To further probe differences in PK which will affect consequent systemic exposure, blood samples were collected over 120 h, and the decay-corrected relative levels of [^64^Cu]-labeled constructs were assessed at each time (Figure 6C). These results demonstrate stark differences in PK between unmodified constructs (ANT4044 IgG1 and HuJ591) in comparison to PK-modified antibodies (ANT4044 IgG1 (H310A, H435Q) and IgG4 (S228P, L235E, H310A, H435Q)). At all time points assayed, unmodified constructs showed elevated levels in circulation, and while these remained at significant levels up to 120 h post-administration (ANT4044 IgG1 at 10%ID/g and HuJ591 at 7%ID/g), constructs unable to undergo FcRn recycling reached unmeasurable levels between 72 and 96 h post-administration. This resulted in a calculated pharmacological half-life of 7.3 and 8.7 h for IgG1 (H310A, H435Q) and IgG4 (S228P, L235E, H310A, H435Q), respectively, while ANT4044 and HuJ591 clearance was unable to be conclusively fit within the 5-day time course assayed. These results are in congruence with the design approach utilized here to reduce circulation time through engineering approaches that selectively inhibit FcRn binding.

The key aim of the work was to engineer constructs that maintain efficient tumor accumulation with a shortened PK profile to reduce systemic exposure. Figure 6D contrasts the measured tumor:blood ratio at 48 h, demonstrating that variants with abolished FcRn binding achieve a greater than 4-fold enhancement in the tumor:blood ratio, congruent with a successful targeted design process. This trend continues at the later time point assayed of 120 h post-injection (Figure S20); however, at this stage, the blood level of FcRn-abolished variants is unmeasurable, making the ratio tend toward meaningless infinity. Together, these results are consistent with successful engineering of PSMA-targeted constructs with beneficial target:systemic exposure profiles, which are likely to be beneficial in therapeutic delivery approaches.

Successful engineering of the antibody platform in this case resulted in clearance from circulation occurring within days rather than weeks of comparable unmodified constructs. At 5 days, only the unmodified constructs are observed at elevated levels in the blood and highly perfused tissues such as the lungs and heart (Figure S20). When a therapeutic radionuclide (e.g., ^177^Lu or ^225^Ac) is substituted for the diagnostic ^64^Cu utilized in this proof-of-concept study, PK-modified antibodies are anticipated to result in significantly reduced systemic exposure, as well as subsequent myelosuppression and hematological toxicity, which is key to the clinical tolerability of TRT. Ideally, this will mitigate some of the difficulties with dose fractionation commonly applied for antibody delivered TRT by enabling higher doses to be administered closer together.

Moreover, the circulating half-life of PK-modified antibodies ANT4044 IgG1 (H310A, H435Q) and IgG4 (S228P, L235E, H310A, H435Q) is actually comparable to that reported for [^177^Lu]-PSMA-617, thereby both limiting hematological toxicities, which is critical for TRT.^29^ However, the route of clearance differs significantly, with small-molecule PSMA radiopharmaceuticals primarily cleared renally, resulting in significant doses to the kidneys and causing potential nephrotoxicity,^6,30^ while the PK-modified antibodies primarily undergo hepatic clearance, which is relatively radiotolerant.^31^ This pharmacokinetic profile, combined with beneficial clearance routes, makes the engineered antibodies described here a promising platform for TRT delivery to prostate cancer.

### Antibody Stability

Antibody constructs designed for application in the clinic require sufficient stability to tolerate distribution and handling prior to administration. Lead construct DOTA-functionalized ANT4044 IgG1 (H310A, H435Q) stability was assessed in 20 mM Na acetate, 150 mM NaCl at pH 5.5, or 200 mM Na acetate at pH 5.5 buffer and compared to PBS (pH 7.1–7.5) alone. Conjugates in each formulation were stored at −20 °C, 25 °C, or 40 °C, and stability was assessed by analytical SEC after 2 or 4 weeks of storage or after 3 cycles of freeze–thaw (Table S5). DOTA-ANT4044 IgG1 (H310A, H435Q) was shown to be stable under all assayed conditions, with ≥90% IgG monomer retained in all cases except at 40 °C for ≥2 weeks in PBS or 4 weeks in acetate buffers, where this dipped to 88–89%. This was associated with an increase in smaller fragments, congruent with slow cleavage at elevated temperatures in all 3 formulations. Comparatively, 3 rounds of freeze–thaw cycling showed no significant impact on the construct. Together, these results demonstrate that the engineering approaches undertaken here have resulted in a stable construct suitable for application as a radiopharmaceutical antibody carrier.

## Conclusions

Targeted radionuclide therapy approaches are appealing for the control of prostate cancer; however, current small molecule and antibody-targeting agents each possess some limitations regarding PK and biodistribution profiles. Herein, we report a bioengineering approach to create a novel antibody platform specifically designed for radiation delivery by selectively modifying PK properties to reduce systemic exposure while retaining tumor binding and accumulation characteristics. In doing so, we describe a new derivative of the clinically validated antibody HuJ591 to produce a fully humanized targeting construct, with the same PSMA specificity but with >3-fold faster clearance of excess radiation from systemic circulation. An improved therapeutic window with limited circulation time may especially suit an alpha-emitting therapy by minimizing the potential toxicity of free/recoiled Ac-225 and its daughter radionuclides. Our results show this engineering approach to be successfully implemented using just two conservative mutations in the Fc region of the antibody, providing complete abrogation of FcRn binding to modulate PK while retaining PSMA binding comparable to the parental antibody and other FcR activity. The novel targeting constructs radiolabeled with ^64^Cu were evaluated in a preclinical prostate cancer xenograft model and resulted in comparable tumor accumulation despite accelerated clearance from circulation within 3–4 days. Together, these results demonstrate a successful approach to produce a novel targeting platform for therapeutic delivery that significantly reduces systemic exposure and aims to mitigate potential hematologic toxicity previously observed with standard antibody-delivered radiation.

## Supplementary Material



## Notes

Variable region sequences, humanization process, antibody expression details (plasmid design, SDS-PAGE results following purification, size exclusion chromatography and thermal stability results), SPR data showing construct antigen binding, DOTA-functionalized species characterization, radiolabeling results, *ex vivo* biodistribution results, and stability data (DOCX)

## Abbreviations

PSMA: prostate-specific membrane antigen
TRT: targeted radionuclide therapy
[^68^Ga]-PSMA-11: gallium Ga-68 gozetotide; Illuccix
PET: positron emission tomography;
[^177^Lu]-PSMA-617: lutetium Lu-177 vipivotide tetraxetan; Pluvicto
PK: pharmacokinetic
*T*_m_: melting temperature
EDTA: ethylenediaminetetraacetic acid
NHS-DOTA: 1,4,7,10-tetraazacyclododecane-1,4,7,10-tetraacetic acid mono-*N*-hydroxysuccinimide ester
ROI: region of interest,
%ID/g: percentage injected dose/g
CDR: complementarity-determining regions
FcRn: neonatal Fc receptor
SPR: surface plasmon resonance
DAR: drug to antibody ratio
