# Supplementary material for: Development of a Novel Engineered Antibody Format for PSMA-Targeted Radionuclide Therapy
Source: Mol Pharm. 2025 May 22;22(7):3666–78. doi: 10.1021/acs.molpharmaceut.4c01193 (PMC12670495; doi:10.1021/acs.molpharmaceut.4c01193)
Supplement: Supplementary file 1 [file mp4c01193_si_001.docx]

Development of a Novel Engineered Antibody format for PSMA Targeted Radionuclide Therapy

Nicholas L. Fletcher,^a,b^ Zachary H. Houston, ^a^ Peter G. Chandler,^c^ Eddie Yan,^c^ Rob Holgate,^d^ Michael Wheatcroft^b,c*^ and Kristofer J Thurecht ^a,b*^

^a^ Centre for Advanced Imaging, Australian Institute for Bioengineering & Nanotechnology, University of Queensland, St. Lucia, QLD 4072, Australia.

^b^ ARC Research Hub for Advanced Manufacture of Targeted Radiopharmaceuticals

^c^ Telix Pharmaceuticals Limited, North Melbourne, Victoria 3051, Australia

^d^ Abzena Limited, Babraham, United Kingdom

# Supplementary Information

## Variable region sequences for ANT4044

**ANT4044 Heavy Chain Variable Region Sequence**


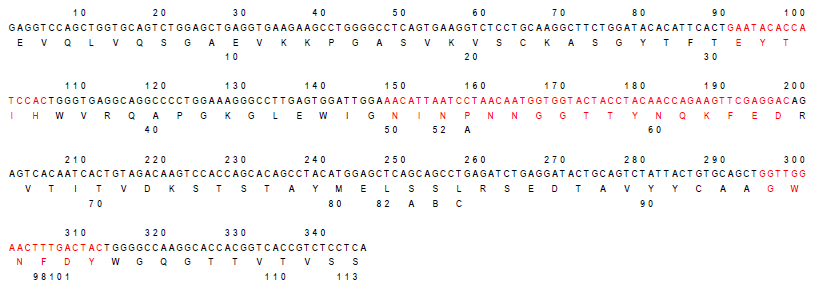


**ANT4044 Light Chain Variable Region Sequence**


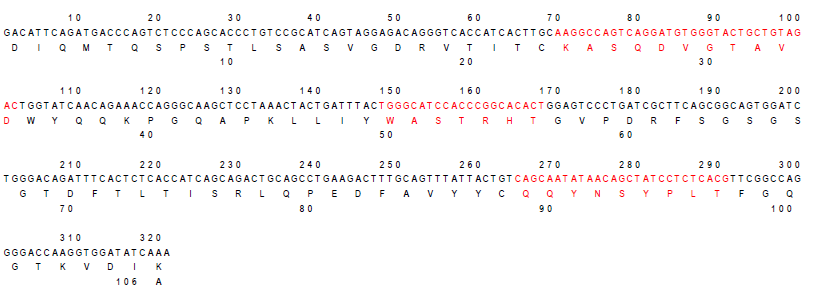


Figure S1. ANT4044 variable region sequences. CDR definitions and protein sequence numbering according to Kabat. CDR nucleotide and protein sequences are highlighted in red

## ANT4044 humanization and deimmunisation using iTope^TM^

The iTope^TM^ software predicts favourable interactions between amino acid side chains of a peptide and specific binding pockets (pocket positions; p1, p4, p6, p7 and p9) within the open-ended binding grooves of 34 human MHC class II alleles. These alleles represent the most common HLA-DR alleles found world-wide with no weighting attributed to those found most prevalently in any ethnic population. Twenty of the alleles contain the “open” p1 configuration and 14 contain the “closed” configuration where glycine at position 83 is replaced by a valine. The location of key binding residues is achieved by the *in silico* generation of 9mer peptides that overlap by eight amino acids spanning the test protein sequence. In-house comparisons with physical MHC class II binding experiments have shown that iTope^TM^ can be used to successfully discriminate with high accuracy between peptides that either bind or do not bind MHC class II molecules. However, the results should be assessed in the light of the fact that all predictive methods for MHC class II binding inherently over-predict the number of T cell epitopes since they do not allow for other important processes during antigen presentation such as protein/peptide processing, recognition by the T cell receptor or T cell tolerance to the peptide.

Analysis of the humanised variant sequences together with HuJ591 using iTope^TM^ was performed with overlapping 9mer peptides (with each overlapping the last peptide by 8

residues) which were tested against each of the 34 MHC class II allotypes. Each 9mer was

scored based on the potential “fit” and interactions with the MHC class II molecules. The peptide scores calculated by the software lie between 0 and 1. Peptides that produced a high mean binding score (>0.55 in the iTope^TM^ scoring function) were highlighted and, if >50% of the MHC class II binding peptides (i.e. 17 out of 34 alleles) had a high binding affinity (score >0.6), such peptides were defined as “promiscuous high affinity” MHC class II binding peptides which are considered a high risk for containing CD4+ T cell epitopes. Promiscuous moderate affinity MHC class II binding peptides bind a high number of alleles (>50%) with a binding score >0.55 (but without a majority >0.6). These criteria were altered in the case of a large aromatic amino acid (i.e. F, W, Y) occurring in the p1 anchor position where the open p1 pocket of 20 of the 34 alleles allows the binding of a large aromatic residue. Where this occurs, a promiscuous peptide is defined as binding to 10 or more of the subset of 20 alleles.

A number of germline promiscuous high and moderate affinity MHC Class II binding ligands

were identified in the parental antibody and designed variants however it is unlikely that

these epitopes have immunogenic potential due to T cell tolerance, and so were excluded from any further analysis. Several non-germline promiscuous high and moderate affinity MHC class II binding ligands were identified in both the heavy and light chain sequences however, it was observed that during the humanisation design process the majority of such epitopes were either removed or showed a reduction in affinity to MHC Class II (summarised in Table 1). However, a subset of epitopes identified in both the V_H_ and Vκ sequences were found to be associated with CDR regions and it is likely that these could not be easily removed without the probability of affecting the ability of the antibody to bind to its antigen.

## Antibody Expression, production and purification


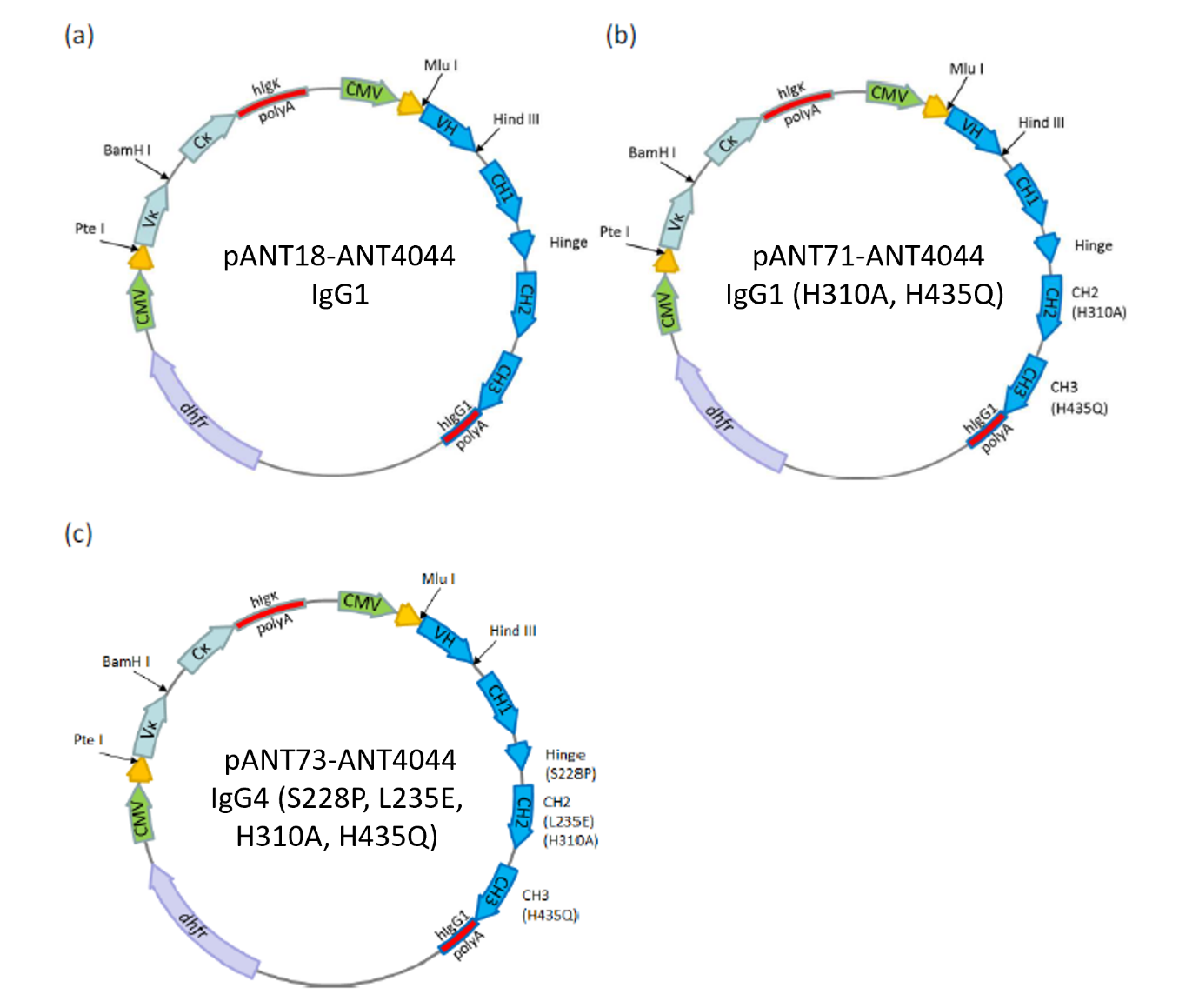


Figure S2. Plasmid maps of Abzena’s pANT human dual expression vectors encoding: (a) ANT4044 IgG1 (pANT18);

(b) ANT4044 IgG1 (H310A, H435Q) (pANT71), and (c) ANT4044 IgG4 (S228P, L235E, H310A, H435Q) (pANT73).


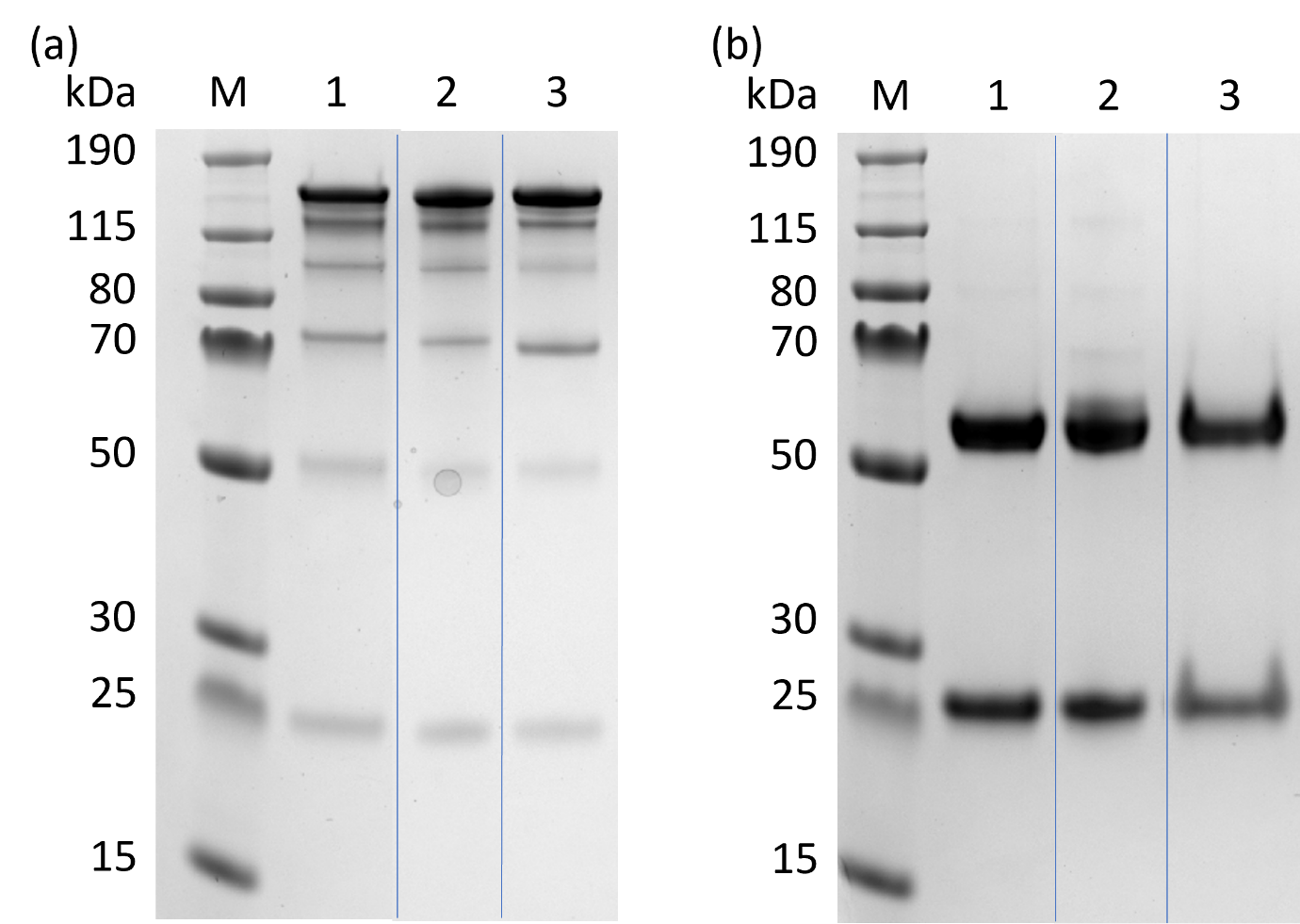


Figure S3. Representative SDS-PAGE gels of Protein A or Protein G purified antibodies following preparative SEC. Molecular weight Marker (M): PageRuler Plus Pre-stained protein standard (ThermoFisher, Loughborough, UK). (a) Samples run under non-reducing conditions; (b) Samples reduced with β-mercaptoethanol. Lane 1: ANT4044 IgG1; lane 2: ANT4044 IgG1 (H310A, H435Q); lane 3: ANT4044 IgG4 (S228P, L235E, H310A, H435Q).


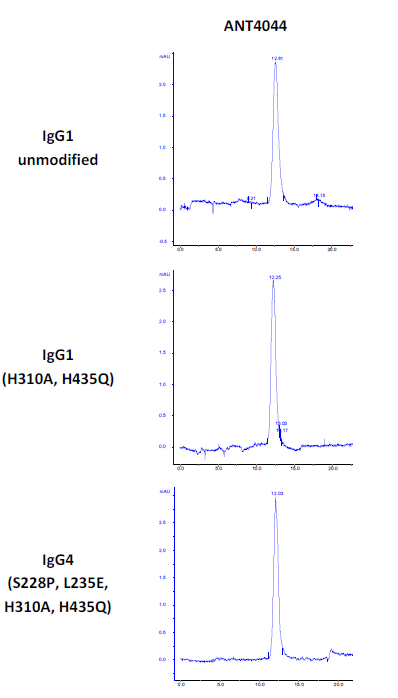


Figure S4 Representative analytical size exclusion chromatograms for each antibody: ANT4044 prepared as IgG1, IgG1 (H310A, H435Q) and IgG4 (S228P, L235E, H310A, H435Q).


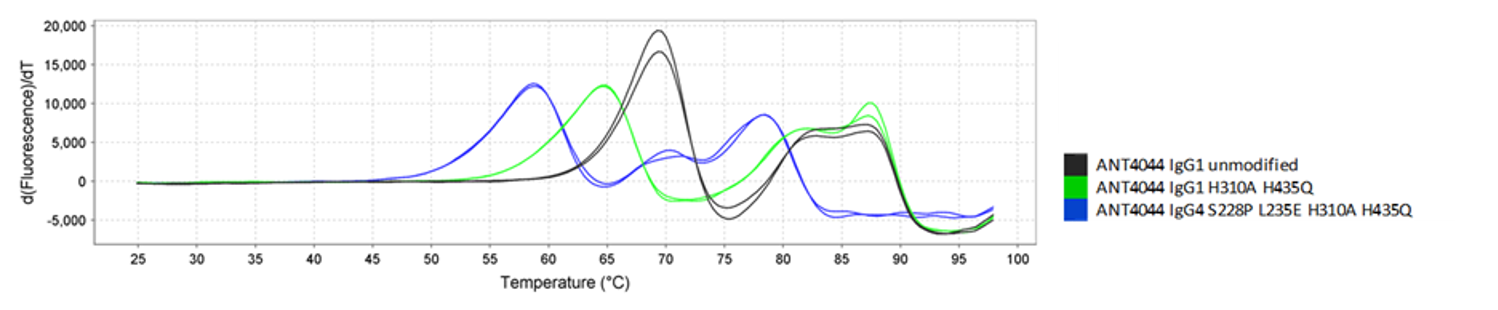


Figure S5. Representative thermostability first derivative melting curves for ANT4044 antibodies expressed in different IgG formats. Antibodies were subjected to a temperature gradient from 25°C to 99°C in duplicate and melting temperatures calculated using protein thermostability software

Table S1. Melting temperatures for the ANT4044 antibodies from production Batches 1 and 2. *For ANT4044 IgG1, the same antibody batch was analysed in each case.

| **IgG Backbone** | Batch 1 | | | Batch 2 | | |
| --- | --- | --- | --- | --- | --- | --- |
|  | T_m1_ | T_m2_ | T_m3_ | T_m1_ | T_m2_ | T_m3_ |
| IgG1 | 69.0 | 87.3 | - | 69.3 | 87.1 | - |
| IgG1 (H310A, H435Q) | 64.6 | 82.1 | 87.3 | 64.7 | 84.7 | 87.6 |
| IgG4 (S228P, L235E, H310A, H435Q) | 59.1 | 70.4 | 78.1 | 58.7 | 70.8 | 78.5 |

## FcR binding studies


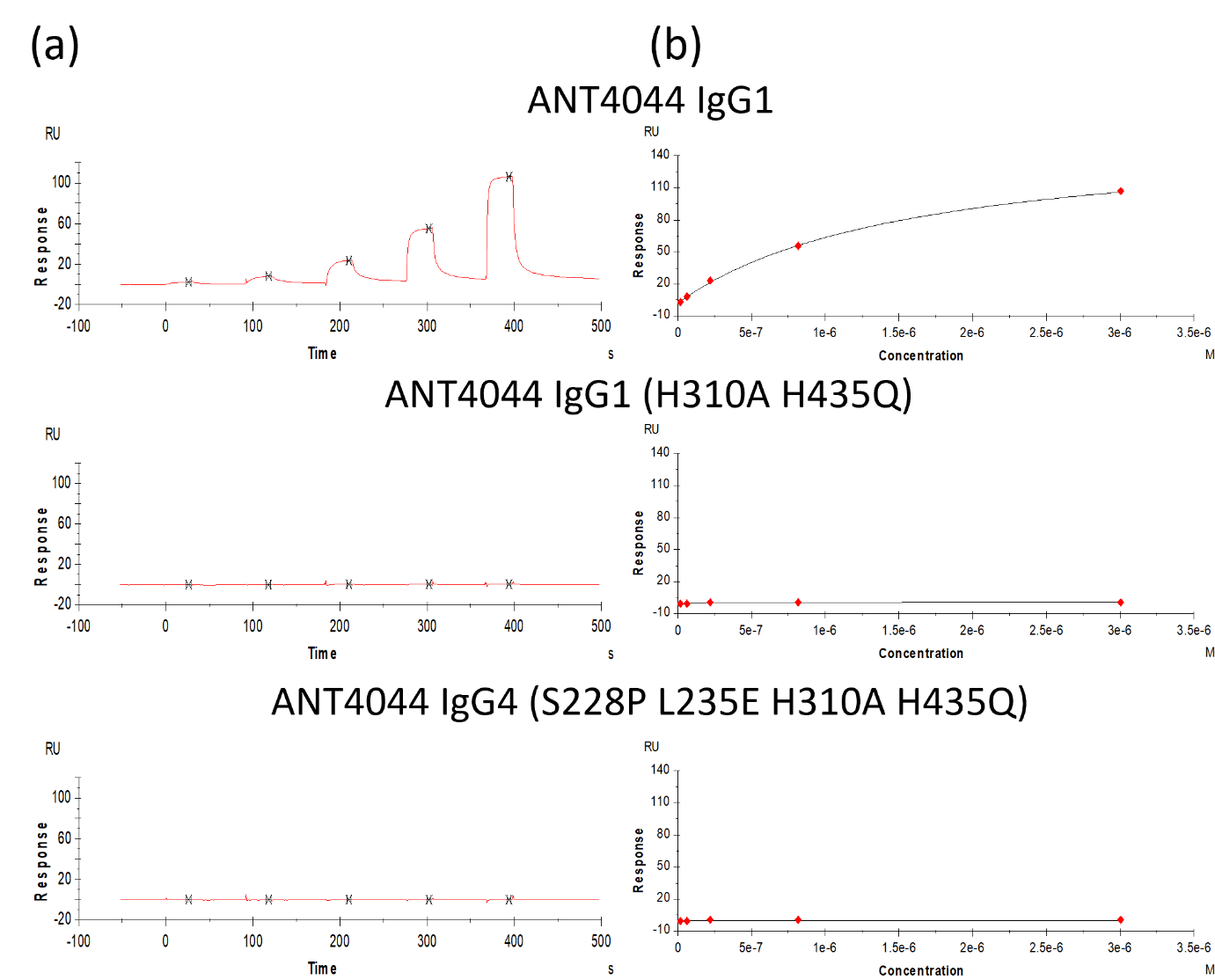


Figure S6. SPR analysis of ANT4044 IgG constructs binding to FcRn at pH 6.0. (a) Steady State affinity sensorgrams of binding of ANT4044 IgG constructs to FcRn. (b) Steady state affinity fitted data for binding to FcRn.


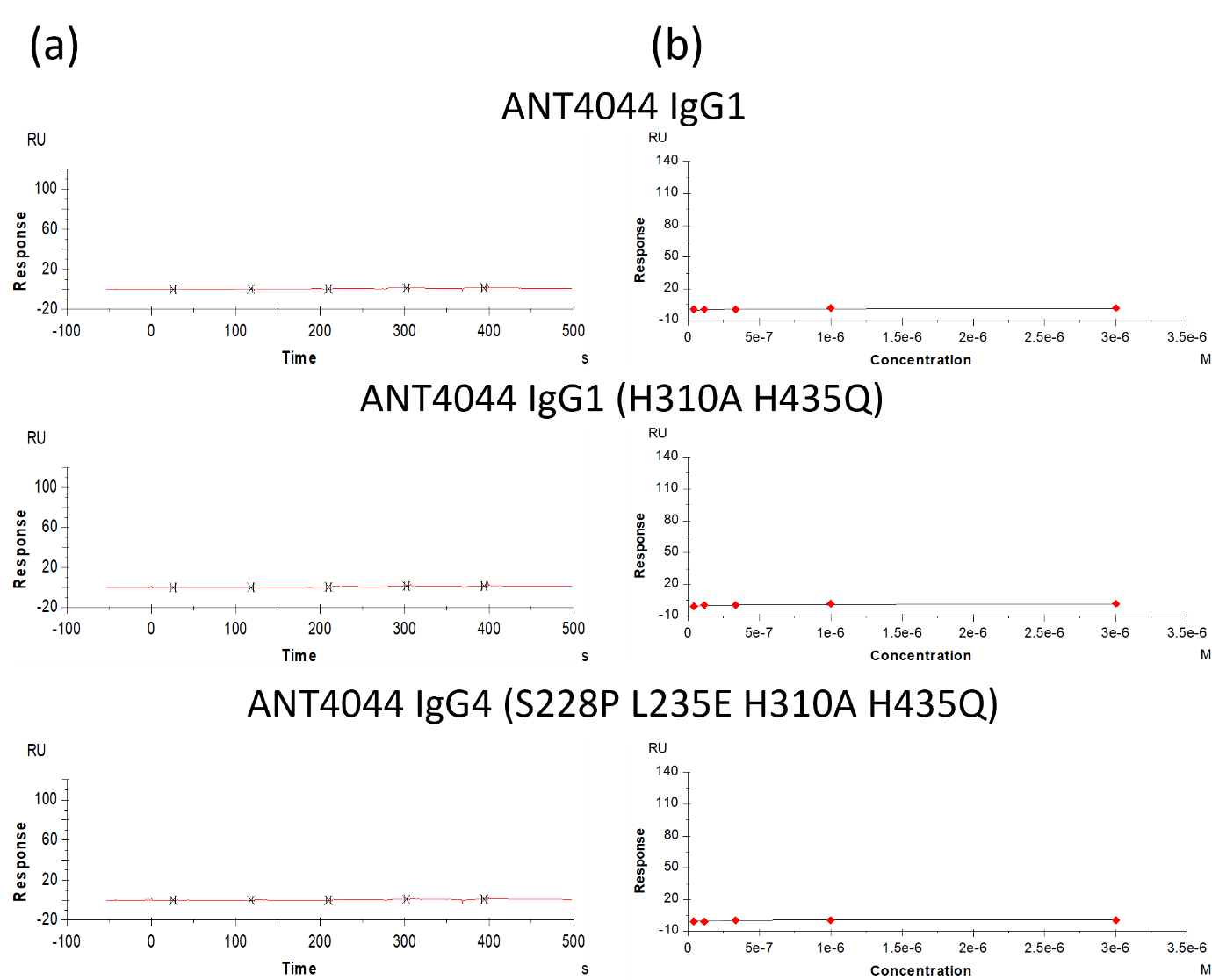


Figure S7. SPR analysis of ANT4044 IgG constructs binding to FcRn at pH 7.4. (a) Steady State affinity sensorgrams of binding of ANT4044 IgG constructs to FcRn. (b) Steady state affinity fitted data for binding to FcRn.

Table S2. List of reagents and parameters used during the Biacore analysis of the binding of antibodies to different FcγRs.

| Name | CD | Binding Affinity | RU loaded | Concentration Range (nM) | Association (s) | Dissociation (s) | Analysis |
| --- | --- | --- | --- | --- | --- | --- | --- |
| FcγRIIIA  ^176Phe^ | CD16A  (176 Phe) | Low | 20 | 98.8-8000 | 45 | 25 | Steady State |
| FcγRIIIA  ^176Val^ | CD16A  (176 Val) | Low | 20 | 98.8-8000 | 45 | 25 | Steady State |
| FcγRIIIB | CD16B | Low | 60 | 296.3-24000 | 30 | 25 | Steady State |
| FcγRIIA  ^167Arg^ | CD32A  (167 Arg) | Low | 60 | 296.3-24000 | 30 | 25 | Steady State |
| FcγRIIA  ^167His^ | CD32A  (167 His) | Low | 30 | 296.3-24000 | 30 | 25 | Steady State |
| FcγRIIB | CD32B | Low | 60 | 296.3-24000 | 30 | 25 | Steady State |
| FcγRI | CD64 | High | 30 | 0.411-33.33 | 200 | 300 | 1:1 Affinity |


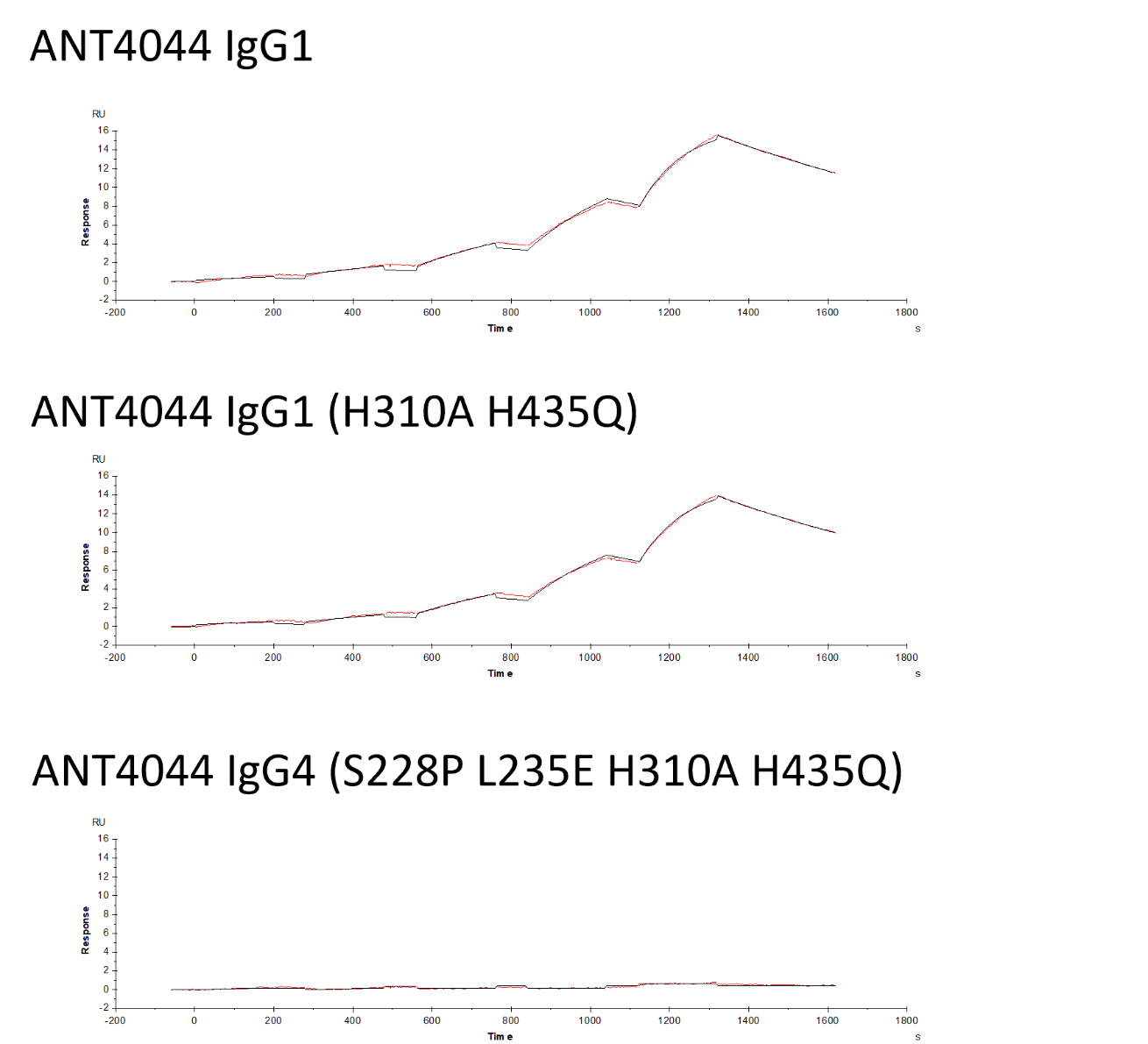


Figure S8. Sensorgrams showing 1:1 binding of: ANT4044 IgG1, ANT4044 IgG1 (H310A, H435Q); and ANT4044 IgG4 (S228P, L235E, H310A, H435Q), to the high affinity FcγRI receptor (CD64). Red line, raw data; Black line, fitted data.


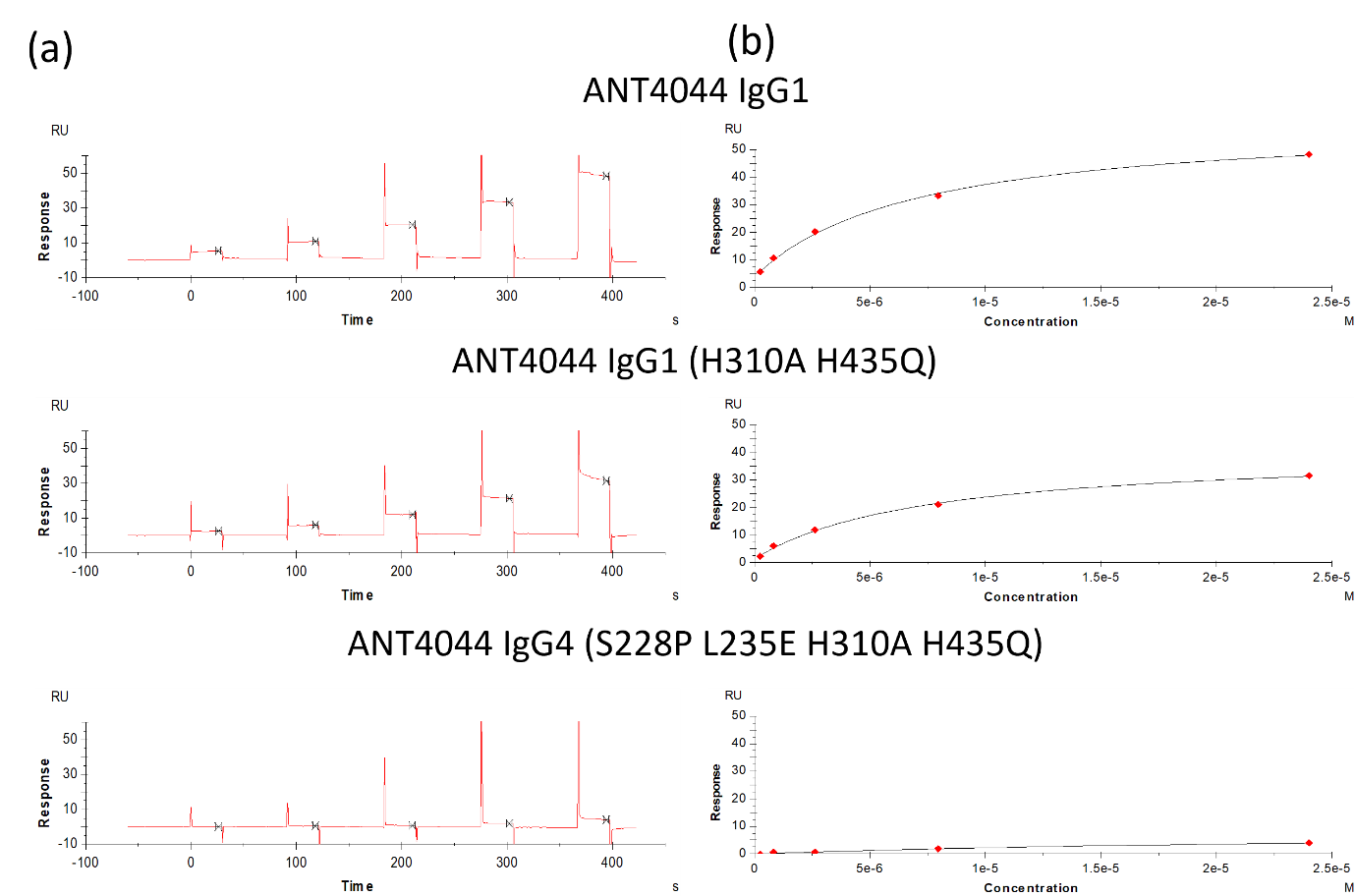


Figure S9. Sensorgrams showing steady state binding (a) and 1:1 binding fitted data (b) of: ANT4044 IgG1, ANT4044 IgG1 (H310A, H435Q); and ANT4044 IgG4 (S228P, L235E, H310A, H435Q), to the low affinity receptor FcγRIIA (CD32A) (His167).


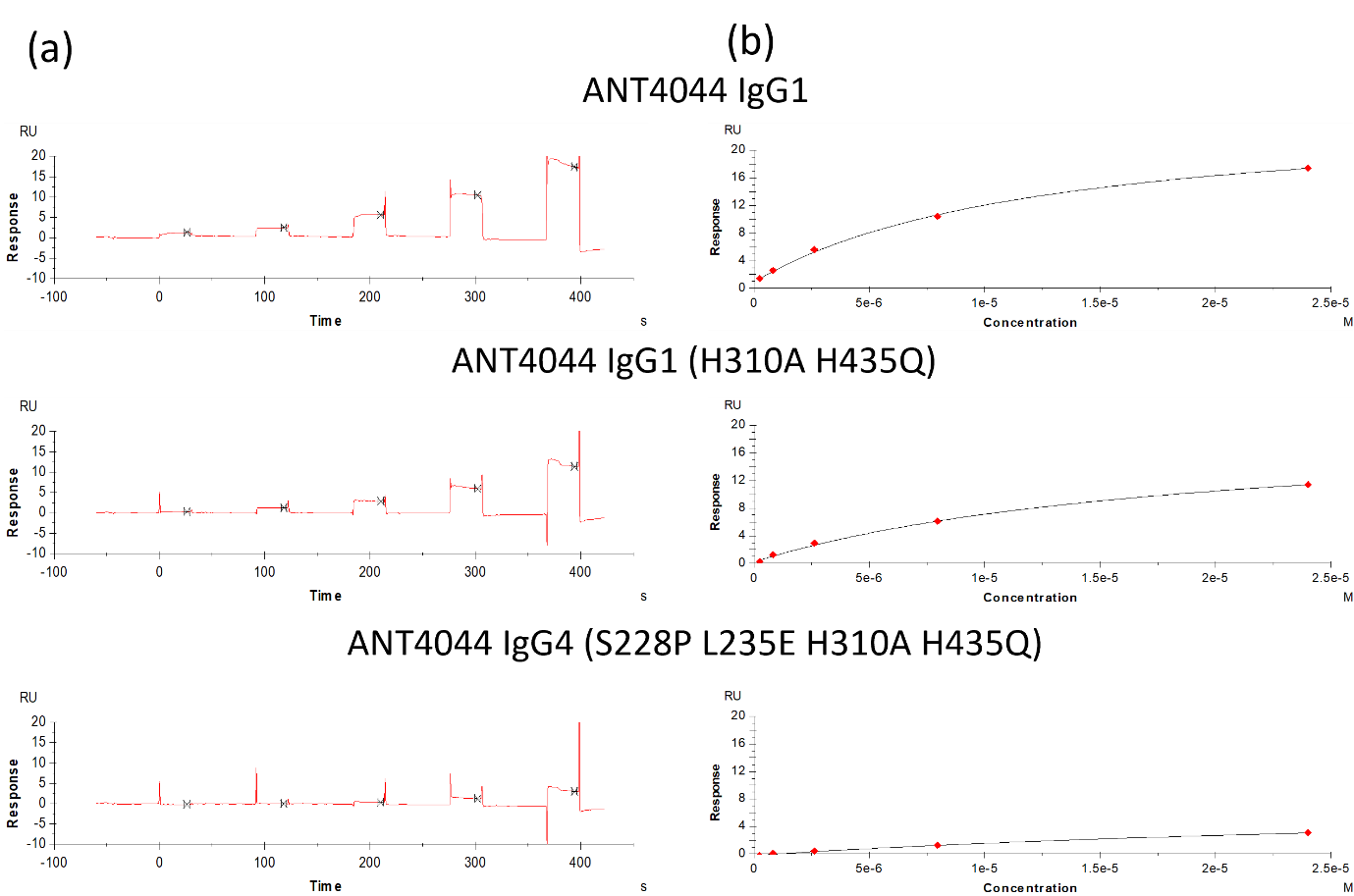


Figure S10. Sensorgrams showing steady state binding (a) and 1:1 binding fitted data (b) of: ANT4044 IgG1, ANT4044 IgG1 (H310A, H435Q); and ANT4044 IgG4 (S228P, L235E, H310A, H435Q), to the low affinity receptor FcγRIIA (CD32A) (Arg167).


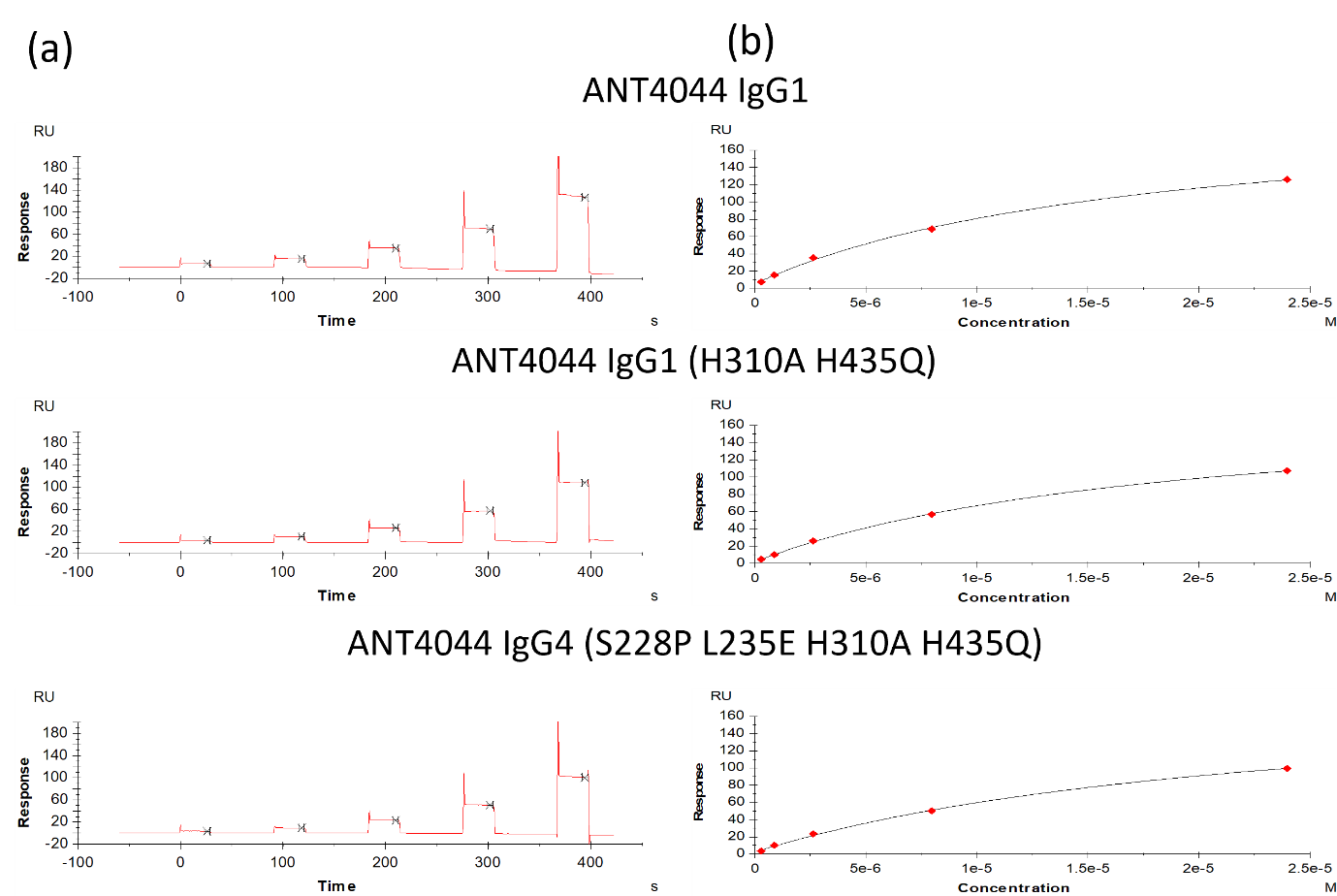


Figure S11. Sensorgrams showing steady state binding (a) and 1:1 binding fitted data (b) of: ANT4044 IgG1, ANT4044 IgG1 (H310A, H435Q); and ANT4044 IgG4 (S228P, L235E, H310A, H435Q), to the low affinity receptor FcγRIIB (CD32B).


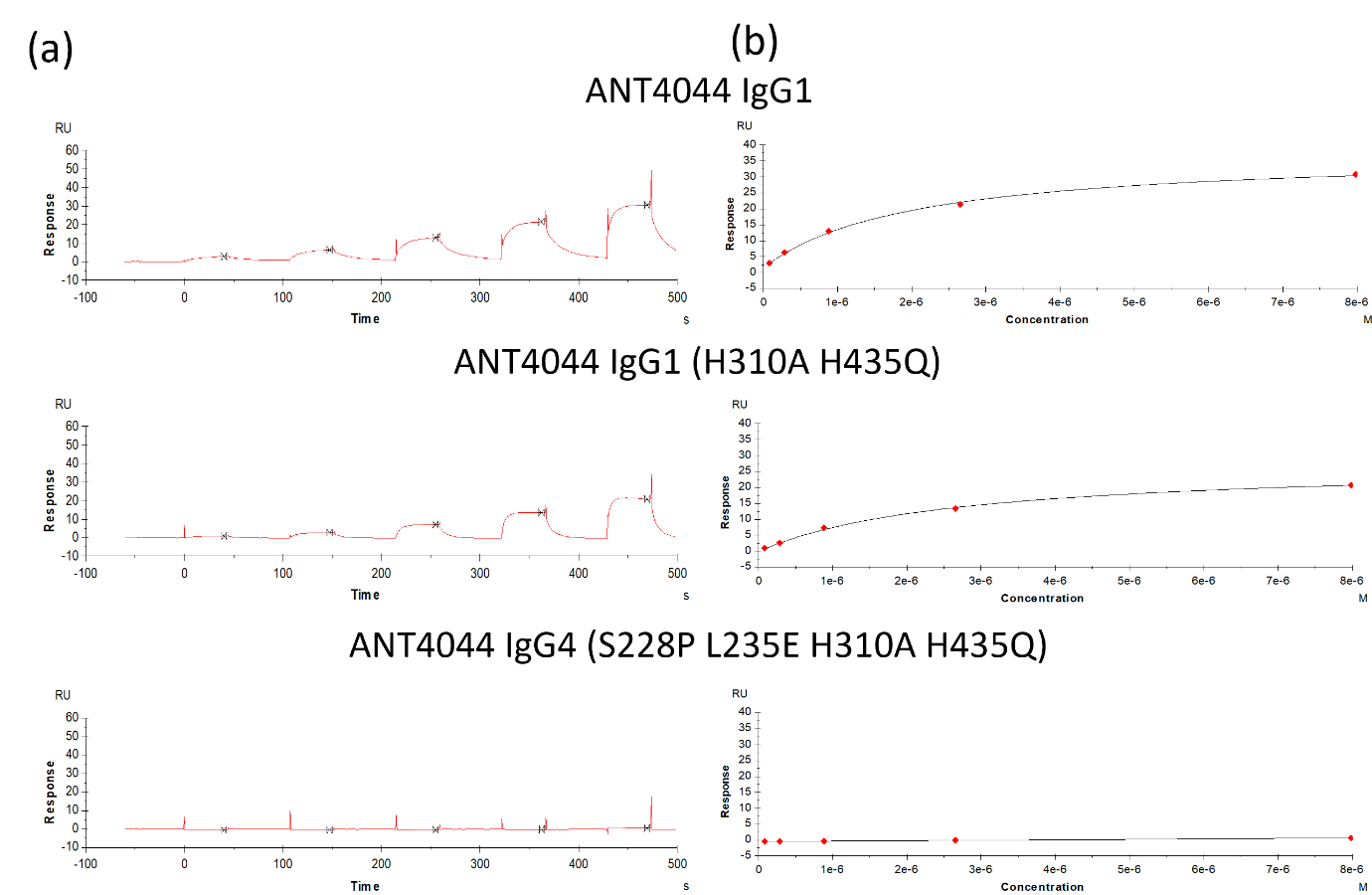


Figure S12. Sensorgrams showing steady state binding (A) and 1:1 binding fitted data (B) of: ANT4044 IgG1, ANT4044 IgG1 (H310A, H435Q); and ANT4044 IgG4 (S228P, L235E, H310A, H435Q), to the low affinity receptor FcγRIIIA (CD16A) (Phe176).


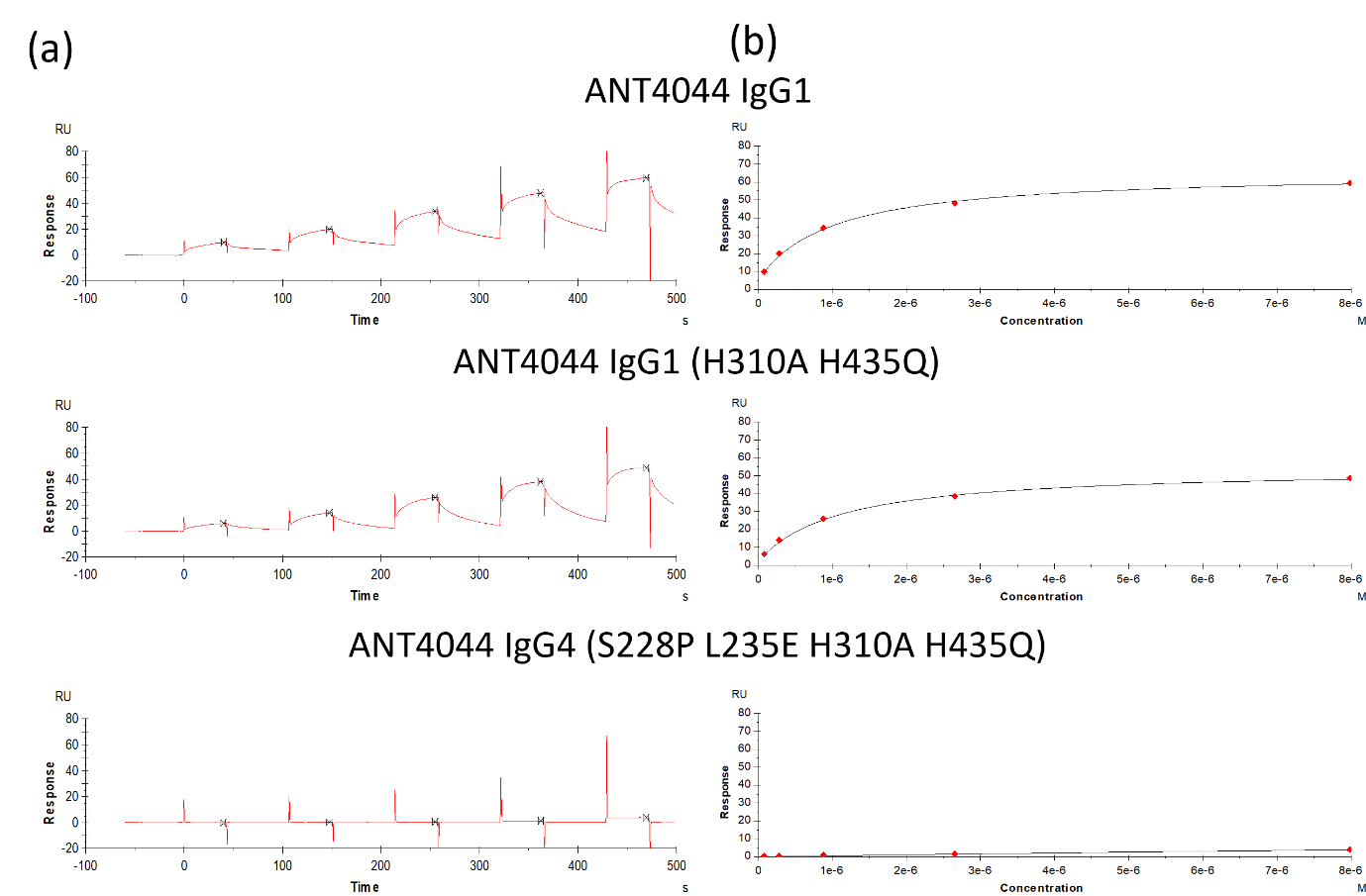


Figure S13. Sensorgrams showing steady state binding (A) and 1:1 binding fitted data (B) of: ANT4044 IgG1, ANT4044 IgG1 (H310A, H435Q); and ANT4044 IgG4 (S228P, L235E, H310A, H435Q), to the low affinity receptor FcγRIIIA (CD16A) (Val176).


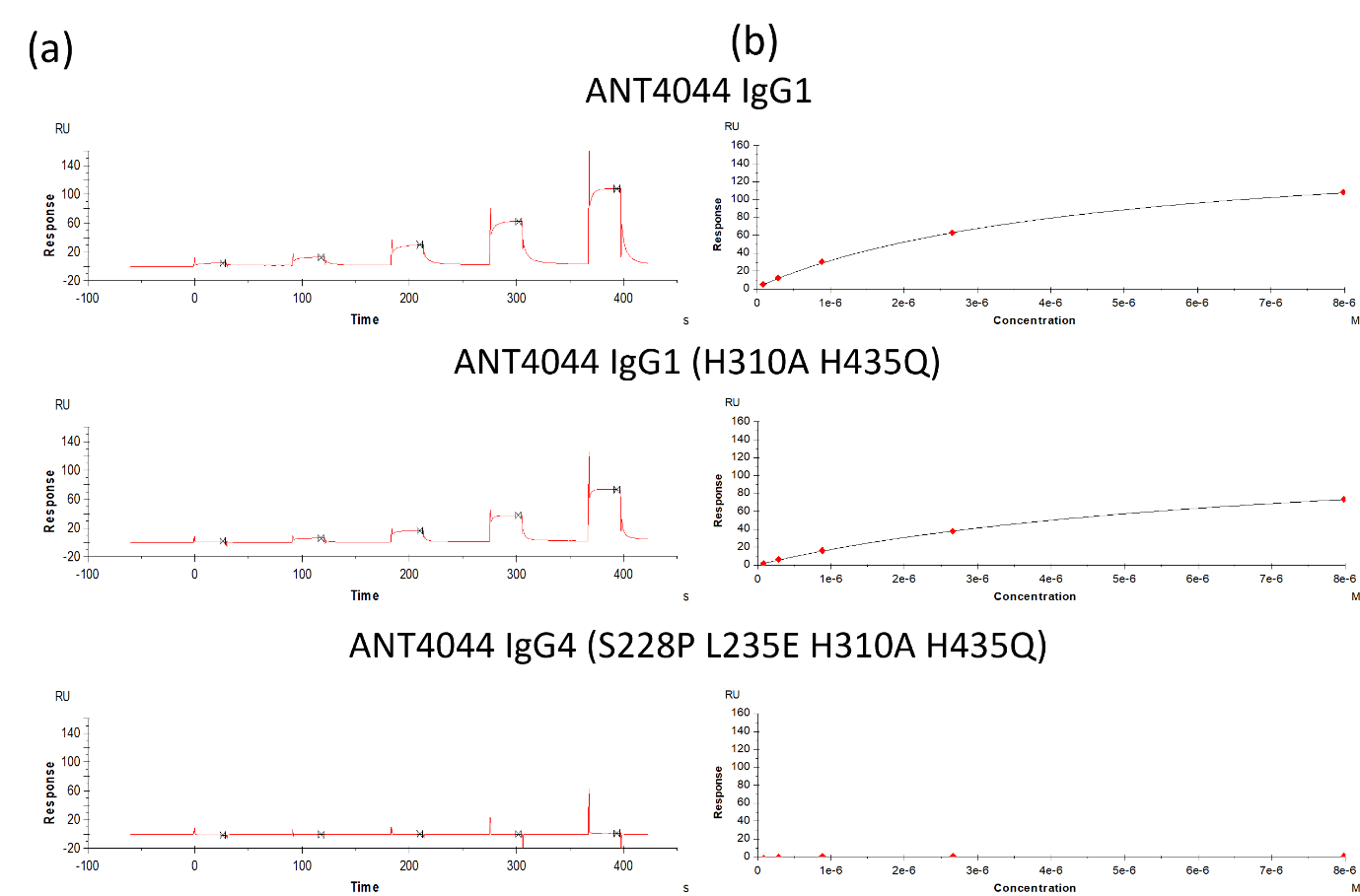


Figure S14. Sensorgrams showing steady state binding (a) and 1:1 binding fitted data (b) of: ANT4044 IgG1, ANT4044 IgG1 (H310A, H435Q); and ANT4044 IgG4 (S228P, L235E, H310A, H435Q), to the low affinity receptor FcγRIIIB (CD16B).

Table S3. Summary batch data of antibody binding to: FcγRI (CD64); FcγRIIA (CD32A) (both His167 and Arg167 allotypes)); FcγRIIB (CD32B); FcγRIIIA (CD16A) (both Phe176 and Val176 allotypes); and FcγRIIIB (CD16B).Values represent calculated mean K_D_ values.

| **IgG Backbone** | Batch | FcγRI (CD64) | FcγRIIA  (CD32A)  His167 | FcγRIIA  (CD32A)  Arg167 | FcγRIIB  (CD32B) | FcγRIIIA  (CD16A)  Phe176 | FcγRIIIA  (CD16A)  Val176 | FcγRIIIB  (CD16B) |
| --- | --- | --- | --- | --- | --- | --- | --- | --- |
| IgG1 | 1 | 4.20 x10^-9^ | 7.04 x10^-6^ | 1.25 x10^-5^ | 1.75 x10^-5^ | 2.01 x10^-6^ | 9.84 x10^-7^ | 6.44 x10^-6^ |
|  | 2 | 5.88 x10^-9^ | 8.36 x10^-6^ | 1.37 x10^-5^ | 1.22 x10^-5^ | 8.87 x10^-7^ | 9.72 x10^-7^ | 4.10 x10^-6^ |
| IgG1 (H310A, H435Q) | 1 | 5.06 x10^-9^ | 7.54 x10^-6^ | 1.90 x10^-5^ | 1.92 x10^-5^ | 2.69 x10^-6^ | 1.09 x10^-6^ | 9.14 x10^-6^ |
|  | 2 | 4.66 x10^-9^ | 6.08 x10^-6^ | 1.40 x10^-5^ | 1.75 x10^-5^ | 1.09 x10^-6^ | 9.59 x10^-7^ | 8.44 x10^-6^ |
| IgG4 (S228P, L235E, H310A, H435Q) | 1 | - | - | - | 2.38 x10^-5^ | - | - | - |
|  | 2 | - | - | - | 2.98 x10^-5^ | - | - | - |


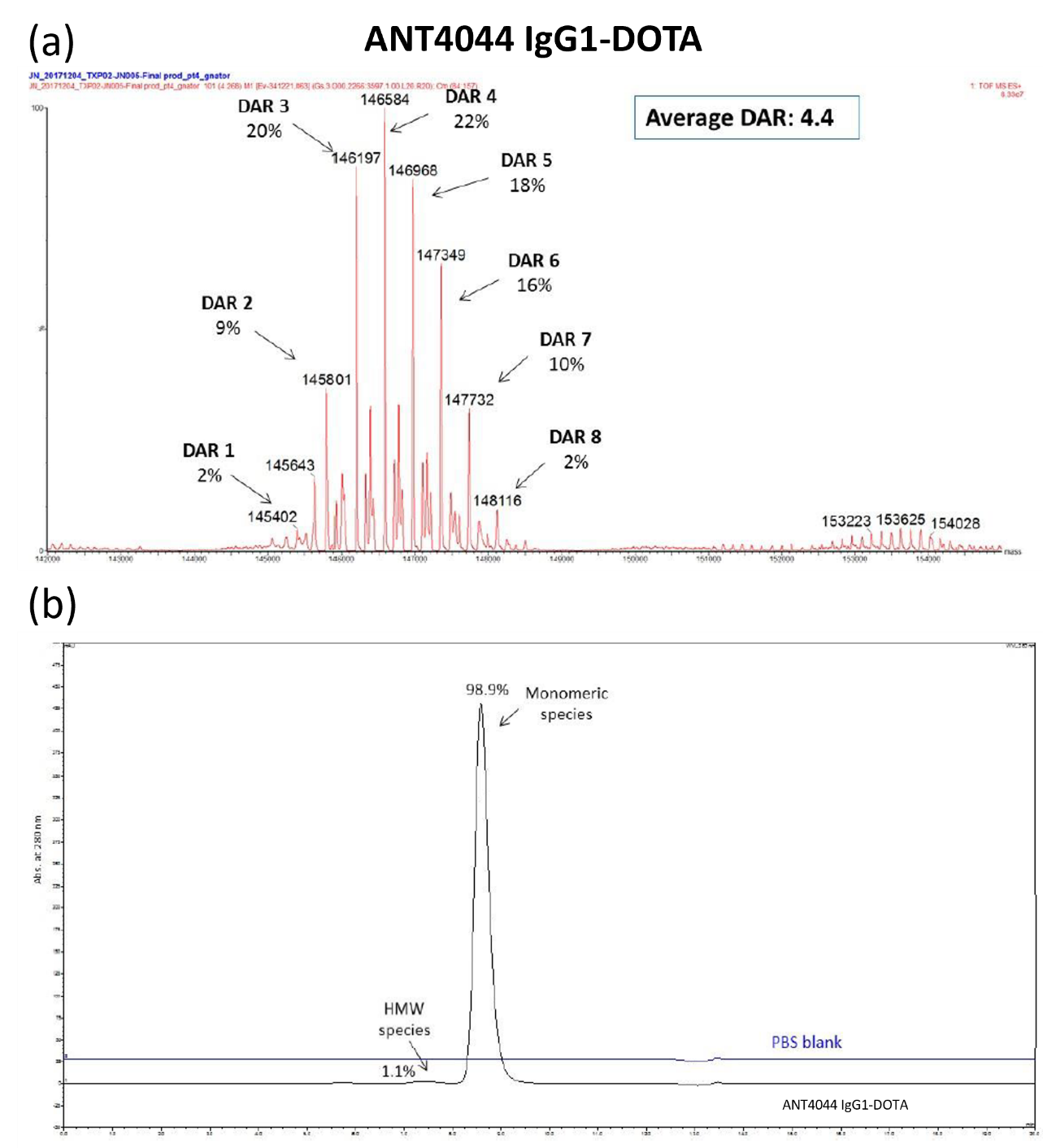


Figure S15. ANT4044-DOTA analysis. (A) LC-MS. (B) analytical SEC.


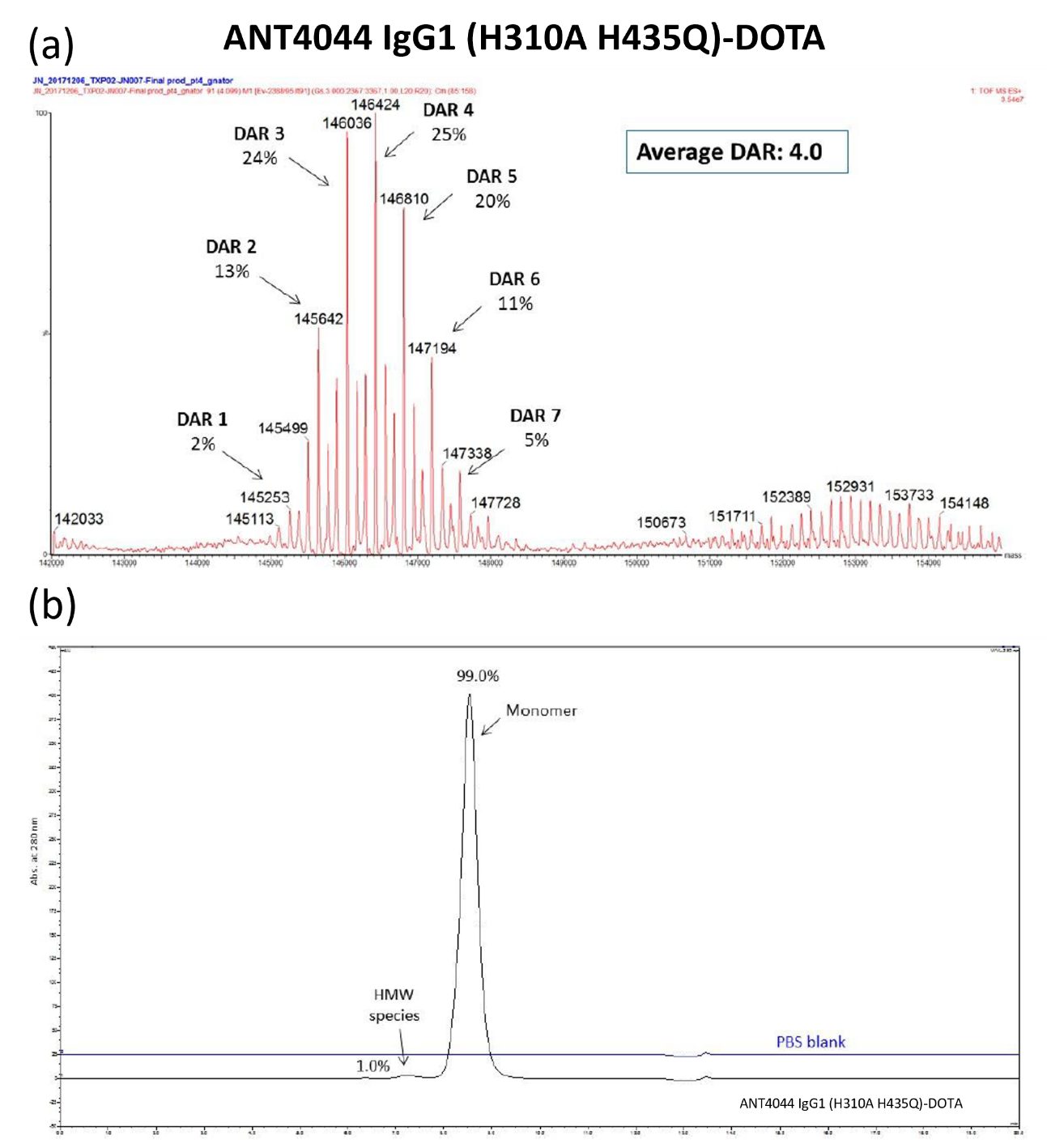


Figure S16. ANT4044-DOTA (H310A, H435Q) analysis. (A) LC-MS. (B) analytical SEC.


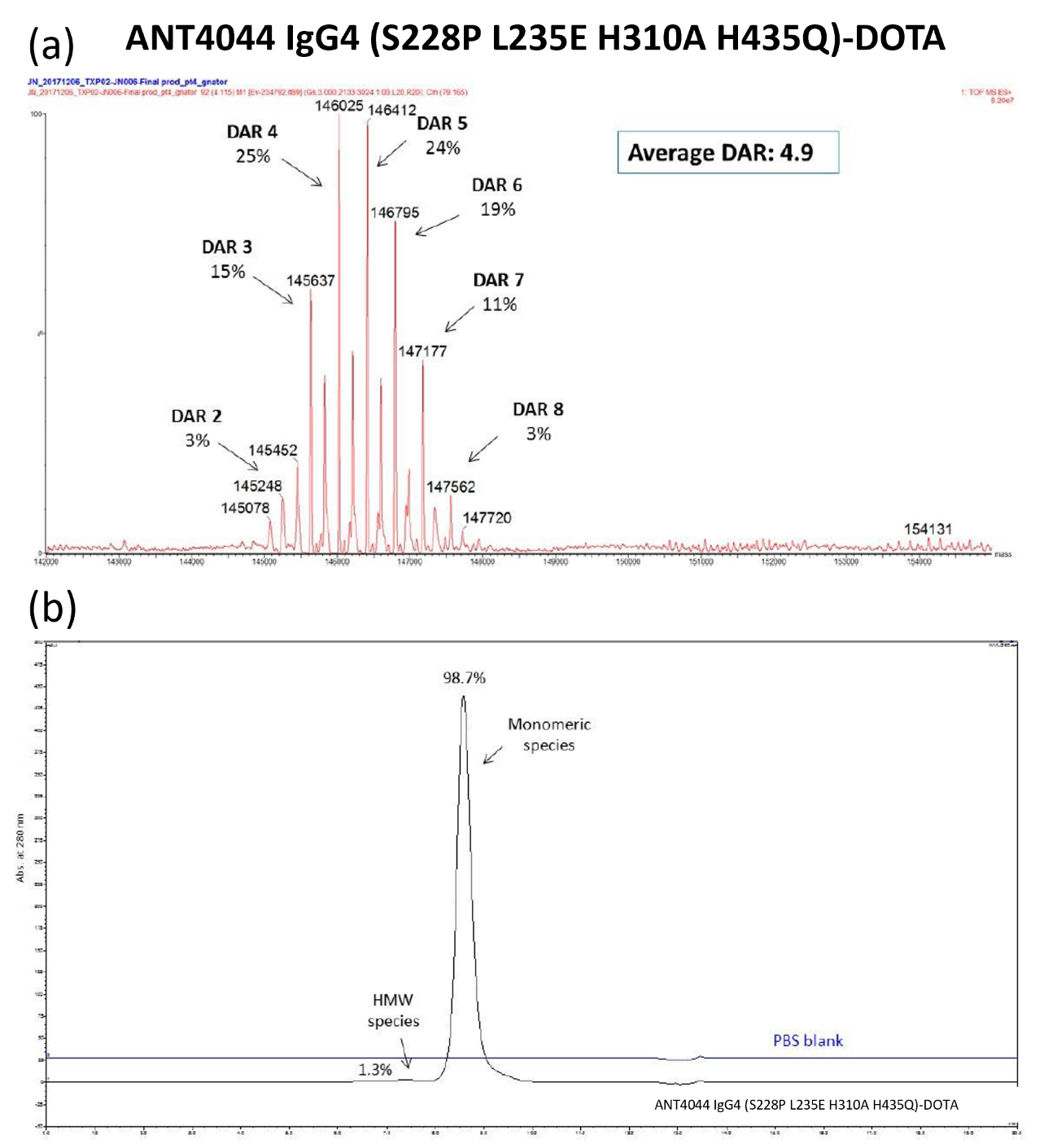


Figure S17. ANT4044-DOTA (S228P, L235E, H310A, H435Q) analysis. (A) LC-MS. (B) analytical SEC.


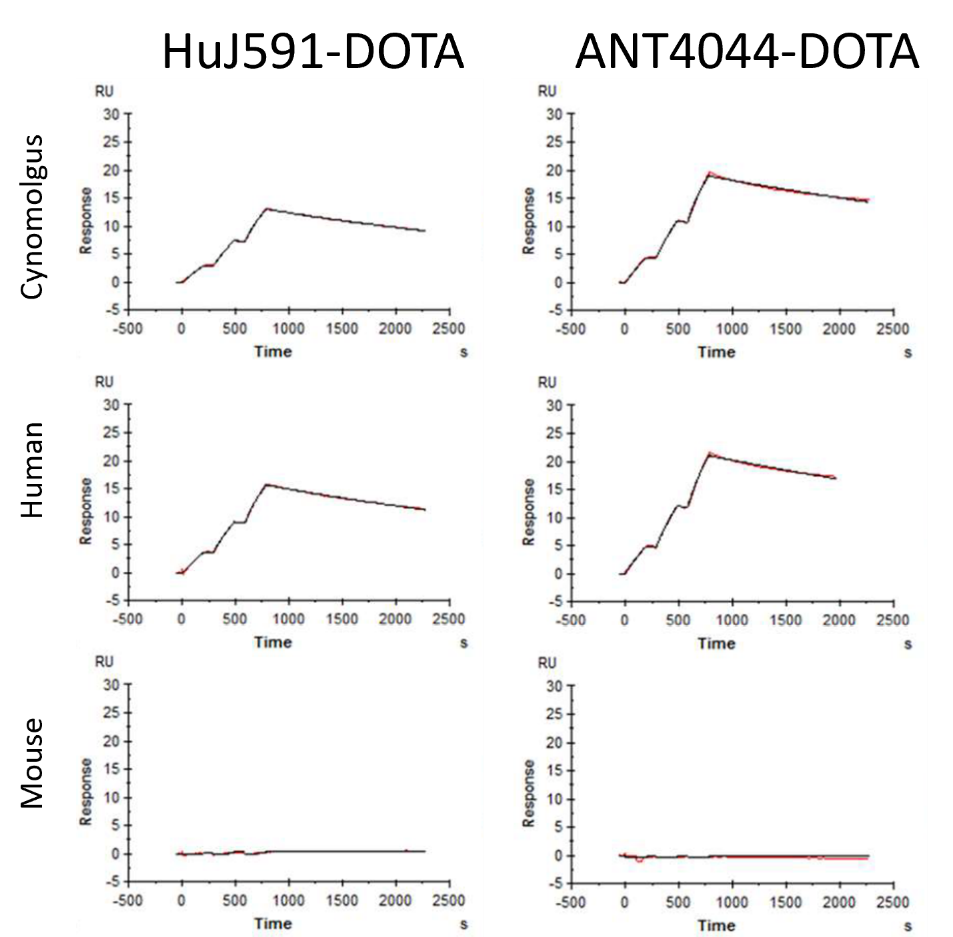


Figure S18. Sensorgrams showing single cycle kinetic analysis of PSMA (Cynomolgus, Human or Mouse) to immobilized HuJ591-DOTA or ANT4044-DOTA

Table S4. Kinetic data for the binding of HuJ591-DOTA and ANT4044-DOTA IgG to PSMA as determined using surface plasmon resonance.

| **Construct** | **PSMA species** | **K_a_ (1/Ms)** | **K_d_  (1/s)** | **K_D_  (M)** | **Chi^2^ (RU^2^)** | **K_a_ (1/Ms)** |
| --- | --- | --- | --- | --- | --- | --- |
| HuJ591-DOTA | Cynomolgus | 6.30 x 10^4^ | 2.40 x 10^-4^ | 3.80 x 10^-9^ | 20.6 | 0.0235 |
|  | Human | 6.96 x 10^4^ | 2.21 x 10^-4^ | 3.18 x 10^-9^ | 23.2 | 0.0431 |
|  | Mouse | - | - | - | - | - |
| ANT4044-DOTA | Cynomolgus | 6.97 x 10^4^ | 1.87 x 10^-4^ | 2.68 x 10^-9^ | 28 | 0.0562 |
|  | Human | 6.63 x 10^4^ | 1.84 x 10^4^ | 2.77 x 10^-9^ | 31.8 | 0.0361 |
|  | Mouse | - | - | - | - | - |


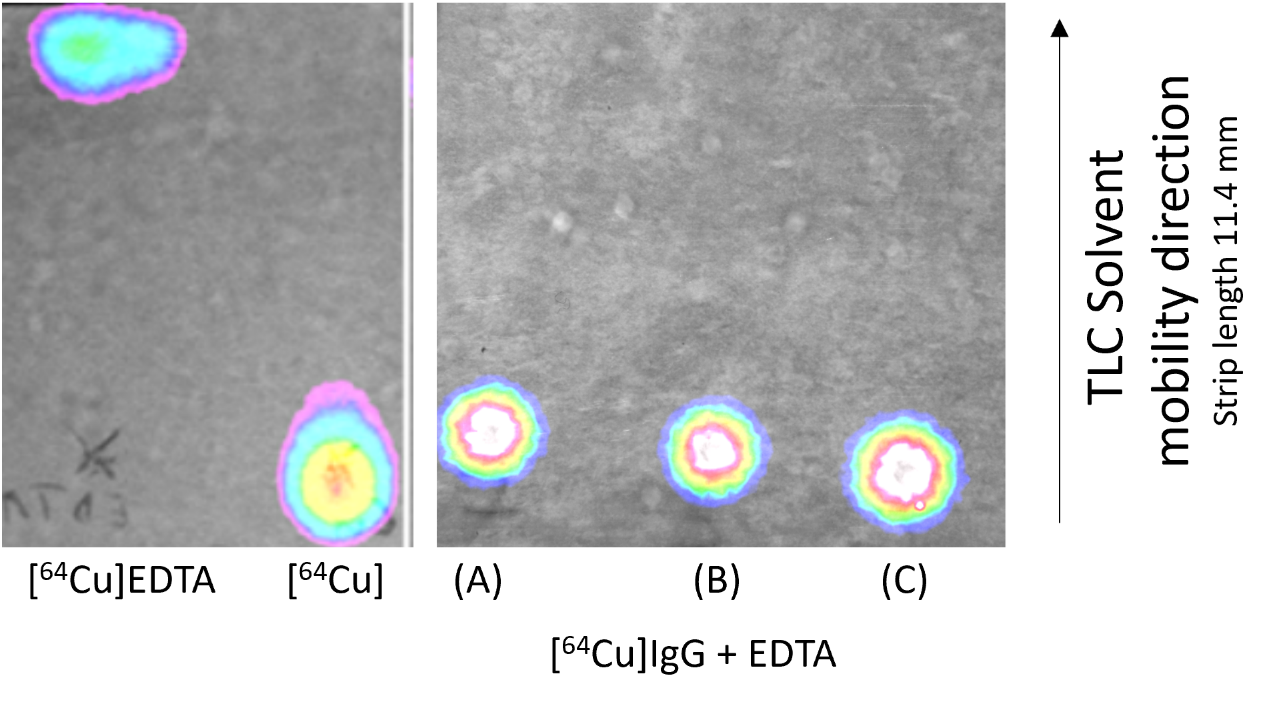


Figure S19. Representative radio-TLC results for controls [^64^Cu] and [^64^Cu]EDTA, as well as [^64^Cu] radiolabelled antibodies; (A) ANT4044 IgG1, (B) ANT4044 IgG1 (H310A, H435Q) and (C) HuJ591 control

Table S5. ANT4044 IgG1 (H310A, H435Q) stability in varied formulations assessed by analytical SEC

| Storage Condition |  | -20 °C | | | 25 °C | | | 40 °C | | | 3 x Freeze-thaw | |
| --- | --- | --- | --- | --- | --- | --- | --- | --- | --- | --- | --- | --- |
|  |  | W0 | W2 | W4 | W0 | W2 | W4 | W0 | W2 | W4 | 0X | 3X |
| 20 mM Na Acetate, pH 5.5, 150 mM NaCl | %HMW | 3.9 | 4.5 | 4.6 | 3.9 | 4.6 | 4.4 | 3.9 | 3.9 | 3.5 | 3.9 | 4.4 |
|  | %monomer | 90.7 | 90.4 | 90.5 | 90.7 | 90.5 | 90.8 | 90.7 | 89.9 | 88.4 | 90.7 | 90.3 |
|  | %LMW | 5.3 | 5.0 | 4.9 | 5.3 | 4.9 | 4.9 | 5.3 | 6.2 | 8.1 | 5.3 | 5.2 |
| 200 mM Na Acetate, pH 5.5 | %HMW | 3.9 | 4.7 | 4.6 | 3.9 | 4.6 | 4.2 | 3.9 | 4.0 | 3.3 | 3.9 | 4.0 |
|  | %monomer | 90.6 | 90.2 | 90.4 | 90.6 | 90.4 | 90.8 | 90.6 | 90.3 | 88.9 | 90.6 | 90.4 |
|  | %LMW | 5.5 | 5.1 | 5.1 | 5.5 | 5.0 | 5.0 | 5.5 | 5.7 | 7.8 | 5.5 | 5.5 |
| PBS pH 7.1-7.5 | %HMW | 4.2 | 5.2 | 5.1 | 4.2 | 5.3 | 5.4 | 4.2 | 5.8 | 5.6 | 4.2 | 4.8 |
|  | %monomer | 90.5 | 89.8 | 90.1 | 90.5 | 89.8 | 89.8 | 90.5 | 89.1 | 89.2 | 90.5 | 90.0 |
|  | %LMW | 5.3 | 5.0 | 4.7 | 5.3 | 4.9 | 4.8 | 5.3 | 5.1 | 5.1 | 5.3 | 5.2 |

Figure S20. Ex vivo biodistribution of [^64^Cu]-labelled ANT4044 variants and HuJ591 at 120 hours post injection. * Denotes P < 0.05, ** Denotes P < 0.01 and *** Denotes P < 0.001 via unpaired t test. Where no significance level is indicated, differences between groups were not statistically significant.
